# New records of cucullanid nematodes from marine fishes off New Caledonia, with descriptions of five new species of *Cucullanus* (Nematoda, Cucullanidae)

**DOI:** 10.1051/parasite/2020030

**Published:** 2020-05-19

**Authors:** František Moravec, Jean-Lou Justine

**Affiliations:** 1 Institute of Parasitology, Biology Centre of the Czech Academy of Sciences Branišovská 31 370 05 České Budějovice Czech Republic; 2 Institut Systématique, Évolution, Biodiversité (ISYEB), Muséum National d’Histoire Naturelle, CNRS, Sorbonne Université, EPHE, Université des Antilles, rue Cuvier CP 51 75005 Paris France

**Keywords:** Nematode parasite, Seuratoidea, Mugiliformes, Perciformes, Tetraodontiformes, South Pacific

## Abstract

Recent examinations of cucullanid nematodes (Cucullanidae) from marine fishes off New Caledonia, collected in the years 2004–2009, revealed the presence of the following five new species of *Cucullanus* Müller, 1777, all parasitic in Perciformes: *Cucullanus variolae* n. sp. from *Variola louti* (type host) and *V*. *albimarginata* (both Serranidae); *Cucullanus acutospiculatus* n. sp. from *Caesio cuning* (Caesionidae); *Cucullanus diagrammae* n. sp. from *Diagramma pictum* (Haemulidae); *Cucullanus parapercidis* n. sp. from *Parapercis xanthozona* (type host) and *P*. *hexophtalma* (both Pinguipedidae); and *Cucullanus petterae* n. sp. from *Epinephelus merra* (type host) and *E*. *fasciatus* (both Serranidae). An additional congeneric species, *Cucullanus bioccai* Orecchia et Paggi, 1987 was recorded from *Mugil cephalus* (Mugilidae, Mugiliformes) (first record in the Pacific Ocean) and *Cucullanus* sp. (only female) was found in *Arothron manilensis* (Tetraodontidae, Tetraodontiformes). Furthermore, two known cucullanid species, *Dichelyne* (*Cucullanellus*) *branchiostegi* (Yamaguti, 1941) in *Branchiostegus wardi* (Malacanthidae, Perciformes) (new host and geographical records) and *Dichelyne* (*Cucullanellus*) *bodiani* Moravec et Justine, 2019 in *Bodianus busellatus* (new host) and *B*. *perditio* (both Labridae, Perciformes), were found; *Dichelyne* (*Cucullanellus*) sp. (only females) coinfecting the latter host may represent an unknown species. Most species are described based on light and electron microscopical studies. The specimens described by Xu et al., 2017 as *Cucullanus bourdini* Petter et Le Bel, 1992 from *Caesio xanthonota* (Caesionidae) in the Taiwan Strait are considered to represent a new species, for which the name *Cucullanus sinensis* n. sp. is proposed.

## Abstract (fr)

L’étude récente de nématodes Cucullanidae de poissons marins du large de la Nouvelle-Calédonie, collectés dans les années 2004-2009, a révélé la présence des cinq nouvelles espèces suivantes de *Cucullanus* Müller, 1777, toutes parasites de Perciformes : *Cucullanus variolae* n. sp. de *Variola louti* (hôte type) et *V. albimarginata* (tous deux Serranidae) ; *Cucullanus acutospiculatus* n. sp. de *Caesio cuning* (Caesionidae) ; *Cucullanus diagrammae* n. sp. de *Diagramma pictum* (Haemulidae) ; *Cucullanus parapercidis* n. sp. de *Parapercis xanthozona* (hôte type) et *P. hexophtalma* (tous deux Pinguipedidae) ; et *Cucullanus petterae* n. sp. d’*Epinephelus merra* (type hôte) et *E. fasciatus* (tous deux Serranidae). Une autre espèce congénérique, *Cucullanus bioccai* Orecchia et Paggi, 1987 est signalée chez *Mugil cephalus* (Mugilidae, Mugiliformes) (première mention dans l’océan Pacifique) et *Cucullanus* sp. (seulement femelle) a été trouvé chez *Arothron manilensis* (Tetraodontidae, Tetraodontiformes). De plus, deux espèces de Cucullanidae connues, *Dichelyne (Cucullanellus) branchiostegi* (Yamaguti, 1941) chez *Branchiostegus wardi* (Malacanthidae, Perciformes) (nouvel hôte et nouvelle mention géographique) et *Dichelyne (Cucullanellus) bodiani* Moravec et Justine, 2019 chez *Bodianus busellatus* (nouvel hôte) et *B. perditio* (tous deux Labridae, Perciformes), ont été trouvées ; *Dichelyne (Cucullanellus)* sp. (seulement femelles) co-infectant ce dernier hôte peut représenter une espèce inconnue. La plupart des espèces sont décrites sur la base d’études au microscope optique et électronique. Les spécimens décrits par Xu et al., 2017 comme *Cucullanus bourdini* Petter et Le Bel, 1992 chez *Caesio xanthonota* (Caesionidae) dans le détroit de Taiwan sont considérés comme représentant une nouvelle espèce, pour laquelle le nom *Cucullanus sinensis* n. sp. est proposé.

## Introduction

A total of eight nominal species of cucullanid nematodes (Cucullanidae) has so far been recorded from marine anguilliform, perciform and tetraodontiform fishes off New Caledonia: *Cucullanus austropacificus* Moravec et Justine, 2018, *C*. *bourdini* Petter et Le Bel, 1992, *C*. *bulbosus* (Lane, 1916), *C*. *epinepheli* Moravec et Justine, 2017, *C*. *gymnothoracis* Moravec et Justine, 2018, *C*. *hansoni* Olsen, 1952, *C*. *incognitus* Moravec et Justine, 2018 and *Dichelyne* (*Cucullanellus*) *bodiani* Moravec et Justine, 2019 [12–16, 23]. Recent examinations of additional materials of cucullanid nematodes from marine fishes off New Caledonia, collected in the years 2004–2009, revealed the presence of five new species of *Cucullanus* Müller, 1777, one previously described species of *Cucullanus*, two known species of *Dichelyne* (*Cucullanellus*) Jägerskiöld, 1902 and two specifically unidentifiable forms (only females available) of each *Cucullanus* and *Dichelyne* (*Cucullanellus*). Results of this study are presented herein.

Three of the newly established species of *Cucullanus* have been described from the male holotypes collected in the respective type host species, whereas morphologically and biometrically very similar females collected from sympatric congeneric hosts are considered to belong to each of them. The conspecificity of females and males in these cases, taking into account the usually wider host specificity in *Cucullanus* spp., is almost certain. Nevertheless, the authors are aware of the fact that it cannot be entirely ruled out, although this is highly improbable, that subsequent studies might show that the females in question do not belong to the newly described species. However, according to the International Code of Zoological Nomenclature, each species is objectively determined by its holotype; the principle of type series helps to solve the situation when specimens of more than one species were included under the same species name. The present authors consider it more reasonable and useful to assign the above-mentioned nematode females to the newly described species than to report them only as *Cucullanus* spp.

## Materials and methods

Fish were caught off New Caledonia by various means; those obtained from the fishmarket in Nouméa were very fresh and were thus probably fished in the near vicinity, the same day. The nematodes for morphological studies were fixed in hot 4% formalin or 70% ethanol. For light microscopical examination (LM), they were cleared with glycerine. Drawings were made with the aid of a Zeiss microscope drawing attachment. Specimens used for scanning electron microscopical examination (SEM) were postfixed in 1% osmium tetroxide (in phosphate buffer), dehydrated through a graded acetone series, critical-pointdried and sputter-coated with gold; they were examined using a JEOL JSM-7401F scanning electron microscope at an accelerating voltage of 4 kV (GB low mode). All measurements are in micrometres, unless otherwise indicated. The fish nomenclature adopted follows FishBase [3].

## Results and discussion

Family Cucullanidae Cobbold, 1864.

### *Cucullanus variolae* n. sp. Figures 1–3

urn:lsid:zoobank.org:act:E11B2721-90C5-48CC-BDF1-2F878600FA79

**Figure 1:**
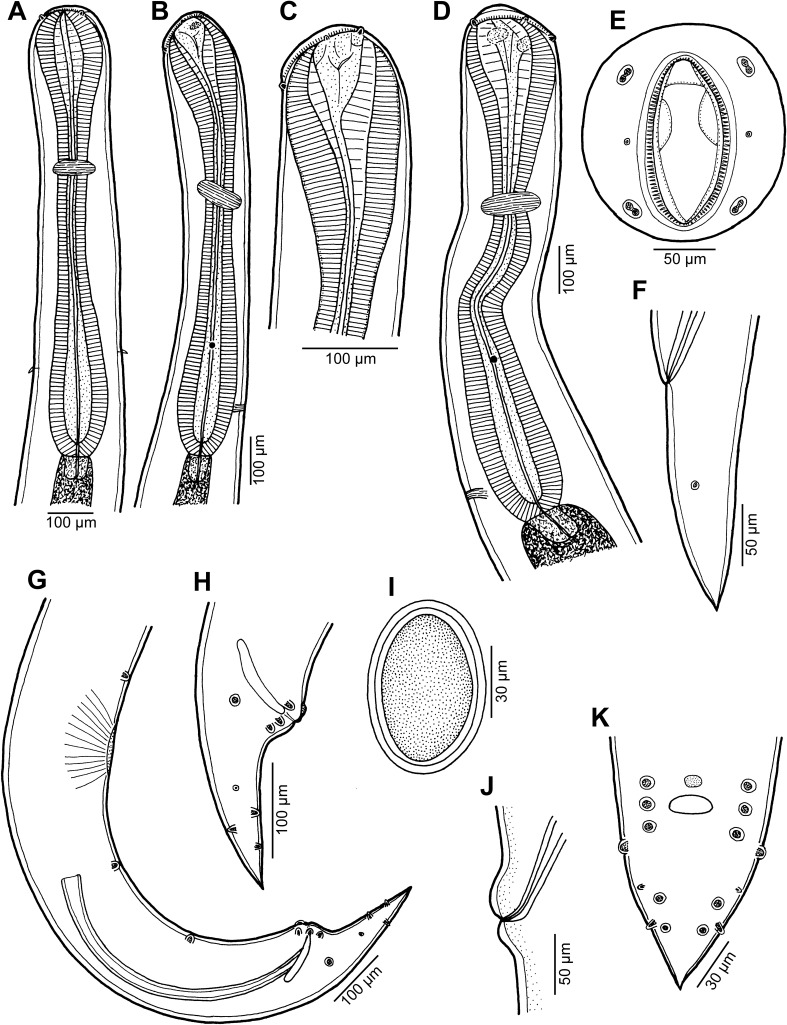
*Cucullanus variolae* n. sp. from *Variola* spp. (A, B) Anterior end of male, dorsoventral and lateral views, respectively; (C) pseudobuccal capsule of male, lateral view; (D) anterior end of female, lateral view; (E) cephalic end, apical view; (F) tail of female, lateral view; (G) posterior end of male, lateral view; (H) tail of male, lateral view; (I) egg; (J) vulva, lateral view; (K) tail of male, ventral view. (A)–(C), (G), (H) and (K) from *V*. *louti*; (D)–(F), (I) and (J) from *V*. *albimarginata*.

Type host: Yellow-edged lyretail *Variola louti* (Forsskål) (Serranidae, Perciformes).

Other host: White-edged lyretail *Variola albimarginata* Baissac (Serranidae, Perciformes).

Site of infection: Intestine.

Type locality: External slope near Récif Toombo, off Nouméa, New Caledonia (collected 21 June 2007) (JNC2198).

Other locality: Récif Le Sournois, off Nouméa, New Caledonia, 22°31,339 S, 166°26,538 E (collected 15 September 2004) (JNC1247).

Prevalence, intensity and details about fish: *V*. *louti*: 1 fish infected/18 fish examined [5]; 2 nematodes. The infected fish, JNC2198, was 475 mm in fork length and 1700 g in weight. *V*. *albimarginata*: 1/2 fish examined [5]; 1 nematode. The infected fish, JNC1247, was 400 mm in fork length and 992 g in weight; this individual fish also harboured *Procamallanus* (*Spirocamallanus*) *variolae* Moravec, Justine et Rigby, 2006.

Deposition of type specimens: Helminthological Collection, Institute of Parasitology, Biology Centre of the Czech Academy of Sciences, České Budějovice, Czech Republic (holotype and allotype mounted on SEM stubs, IPCAS N–1217).

Etymology: The specific name of this nematode relates to the genitive form of the generic name of the hosts.

#### Description

**Figure 2:**
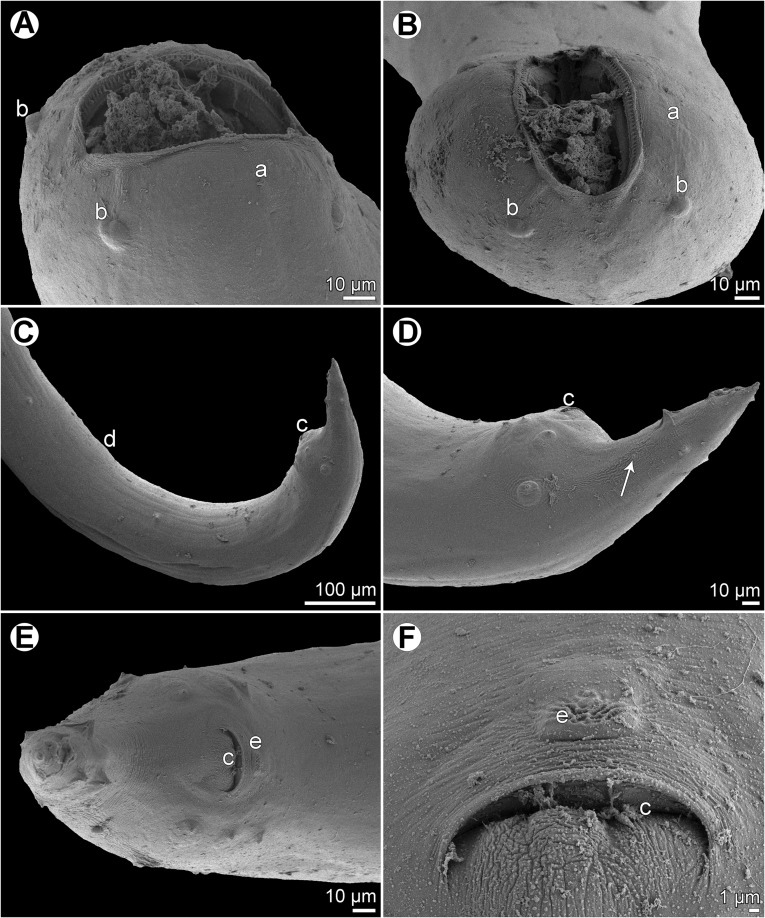
*Cucullanus variolae* n. sp., scanning electron micrographs of male from *V*. *louti*. (A, B) Cephalic end, sublateral and subapical views, respectively; (C) posterior end of body, lateral view; (D) tail, lateral view (arrow indicates phasmid); (E) tail, ventral view; (F) median precloacal elevation, ventral view. (a) Amphid; (b) cephalic papilla; (c) cloaca; (d) ventral sucker; (e) median precloacal elevation.

**Figure 3:**
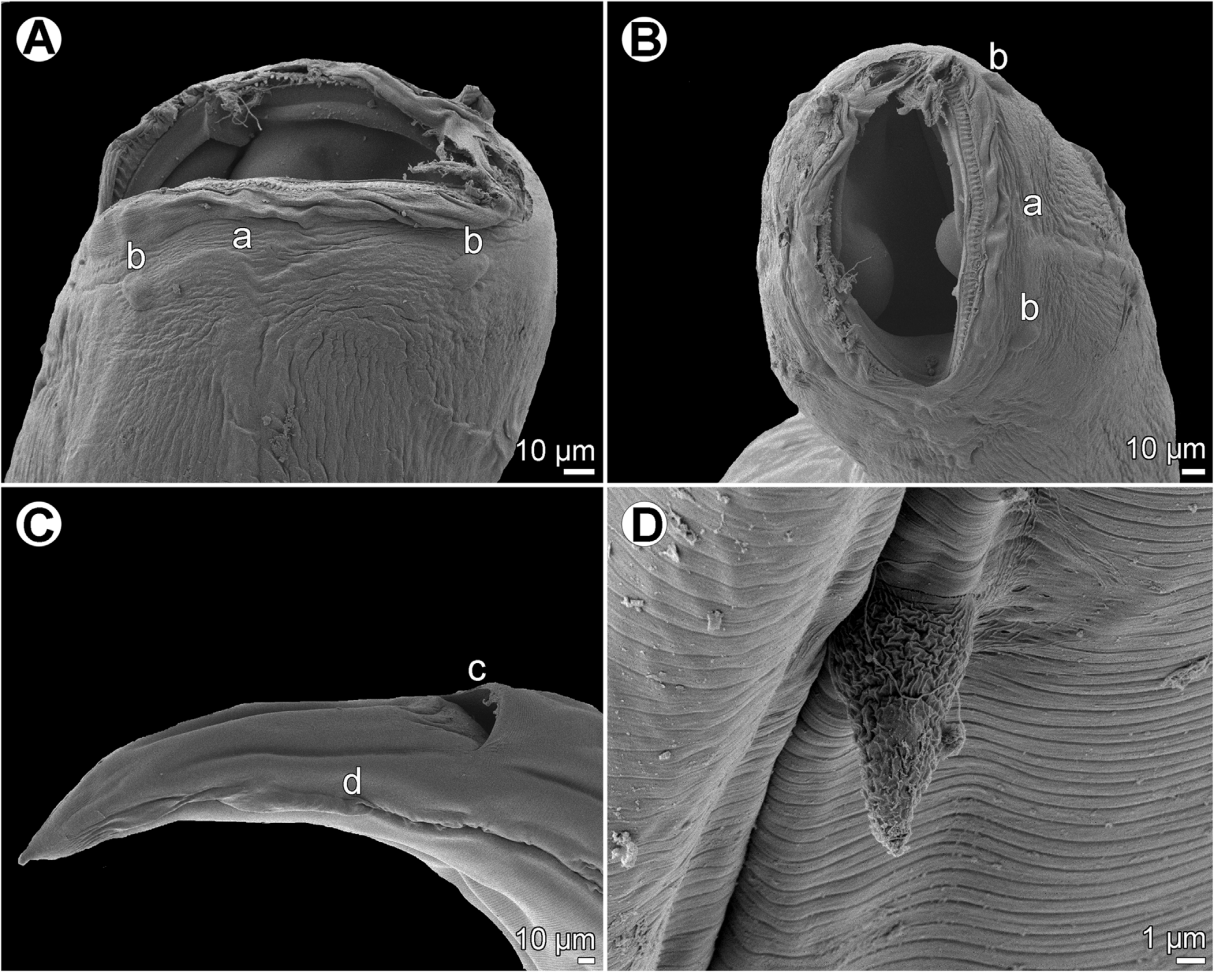
*Cucullanus variolae* n. sp., scanning electron micrographs of female from *V*. *albimarginata*. (A, B) Cephalic end, lateral and apical views, respectively; (C) tail of juvenile specimen, lateral view; (D) deirid. (a) Amphid; (b) cephalic papilla; (c) anus; (d) phasmid.

*General*: Small nematodes with whitish, elongate body. Lateral alae absent. Cephalic end slightly asymmetrical in lateral view (Figs. 1B, 1D). Oral aperture dorsoventrally elongate, surrounded by raised narrow membranous ala (collarette) supported by row of c. 100 minute basal teeth (Figs. 1E, 2A, 2B, 3A, 3B). Four submedian cephalic double papillae and pair of lateral amphids present (Figs. 1E, 2A, 2B, 3A, 3B). Oesophagus muscular, expanded at anterior end to form elongate pseudobuccal capsule (oesophastome) slightly asymmetrical in lateral view; posterior part of oesophagus also expanded, somewhat narrower than oesophastome in lateral view (Figs. 1B, 1D). Oesophagus opens into intestine through large valve. Nerve ring encircles oesophagus approximately at 40% of its length. Deirids small, pointed, situated approximately at mid-way between nerve ring and oesophagointestinal junction or somewhat posterior to it (Figs. 1A, 1B, 1D, 3D). Postdeirids not found. Excretory pore situated short distance anterior to end of oesophagus (Figs. 1B, 1D). Tail of both sexes conical, pointed.

*Male* (1 specimen from *V*. *louti*, holotype): Length of body 9.25 mm, maximum width 218; width at level of oesophastome 150, at middle of oesophagus 136. Length of entire oesophagus 966, representing 10% of whole body length; length of oesophastome 299, its width 136; minimum width of oesophagus 68; maximum width of posterior part of oesophagus 122. Distance of nerve ring from anterior extremity 394, representing 41% of oesophageal length. Slightly asymmetrical deirids and excretory pore 802/843 and 843, respectively, from anterior end of body. Posterior end of body curved ventrally. Ventral sucker present (Figs. 1G, 2C). Cloacal region slightly elevated. Small median, transversely oval cuticular elevation present anterior to cloacal opening (Figs. 1K, 2E, 2F); posterior cloacal lip with rugged surface (Fig. 2F). Spicules equal, 510 long, representing 5.5% of body length. Gubernaculum well sclerotized, 105 long, rod-like in lateral view and Y-shaped in ventral view (Figs. 1G, 1H). Caudal papillae 10 pairs: 4 pairs of subventral preanal papillae, 1 pair of subventral adanal papillae and 5 pairs of postanal papillae (3 subventral and 2 lateral and dorsolateral); second pair of preanals rather far posterior to ventral sucker; last preanal, adanal and first postanal pairs of subventrals close to each other; first lateral pair of postanals slightly posterior to level of cloacal aperture; papillae of dorsolateral postanal pair slightly anterior to level of last pair of subventrals (Figs. 1G, 1H, 1K, 2C–2E). Pair of small lateral phasmids somewhat anterior to level of second subventral pair of postanal papillae (Figs. 1G, 1H, 1K, 2D). Length of tail 190 (Figs. 1G, 1H, 1K, 2C, 2D).

*Female* (1 ovigerous specimen from *V*. *albimarginata*, allotype): Length of body 12.83 mm, maximum width 340; width at level of oesophastome 231, at middle of oesophagus 231. Length of entire oesophagus 1.14 mm, representing 9% of whole body length; length of oesophastome 381, its width 218; minimum width of oesophagus 82; maximum width of posterior part of oesophagus 163 (Fig. 1D). Distance of nerve ring from anterior extremity 462, representing 40% of oesophageal length. Deirids and excretory pore 775 and 1088, respectively, from anterior end of body (Fig. 1D). Vulva postequatorial, 7.25 mm from anterior extremity, at 57% of body length; vulval lips elevated (Fig. 1J). Vagina directed anteriorly from vulva. Uteri opposed. Fully developed eggs elongate-oval, thin-walled, size 75–81 × 51–54, with uncleaved contents (Fig. 1I). Tail 394 long, with pointed tip; small lateral phasmids situated approximately at its middle (Figs. 1F, 3C).

#### Remarks

Because of taxonomic problems concerning numerous species of *Cucullanus* due to their rather uniform morphology and often inadequate descriptions, a detailed comparison among all of them is impossible [18]. Therefore, these parasites are mostly dealt with according to their host groups or the geographical region of their distribution, e.g. [14, 17, 22]. At present, only two valid nominal species of *Cucullanus* parasitizing the Serranidae are known [14]: *C*. *mycteropercae* Mejía-Madrid et Guillén-Hernández, 2011 from *Mycteroperca bonaci* Poey from off the coast of Yucatán, Mexico and *C*. *epinepheli* from *Epinephelus chlorostigma* (Valenciennes) off New Caledonia [7, 14]. However, both *C*. *mycteropercae* and *C*. *epinepheli* possess a distinct posterior outgrowth on the elevated anterior cloacal lip, which is absent in the new species, and the precloacal median elevation of *C*. *mycteropercae* and *C*. *epinepheli* has one or two small papillae, respectively (*vs.* no papillae present on the precloacal median elevation in the new species).

Moreover, in contrast to *C*. *variolae* n. sp., *C*. *epinepheli* is characterized by the presence (*vs.* absence) of narrow lateral cervical alae, the posterior part of the oesophagus broader (*vs.* narrower) than the pseudobuccal capsule, longer spicules (748–789 μm *vs.* 510 μm) representing 9–10% (*vs.* 5.5%) of the body length and the excretory pore located somewhat posterior (*vs.* anterior) to the level of the posterior end of the oesophagus. *Cucullanus mycteropercae* also differs from the new species in the presence (*vs.* absence) of postdeirids, the more posterior location of deirids relative to the level of the oesophago-intestinal junction and in the excretory pore situated distinctly posterior (*vs.* anterior) to the oesophagus end. In addition, *C*. *variolae* n. sp. differs from both these species in the genus of fish hosts (*Variola* Swainson *vs. Epinephelus* Bloch or *Mycteroperca* Gill) and from *C*. *mycteropercae* also in the geographical region (South Pacific Ocean *vs.* North Atlantic Ocean).

*Cucullanus variolae* n. sp. was previously mentioned as an unidentified *Cucullanus* sp. in *V*. *louti* and *V*. *albimarginata* off New Caledonia [5, 14].

### *Cucullanus acutospiculatus* n. sp. Figures 4–6

urn:lsid:zoobank.org:act:C3CA3D36-CF41-40D8-B989-2CB259AC3789

**Figure 4:**
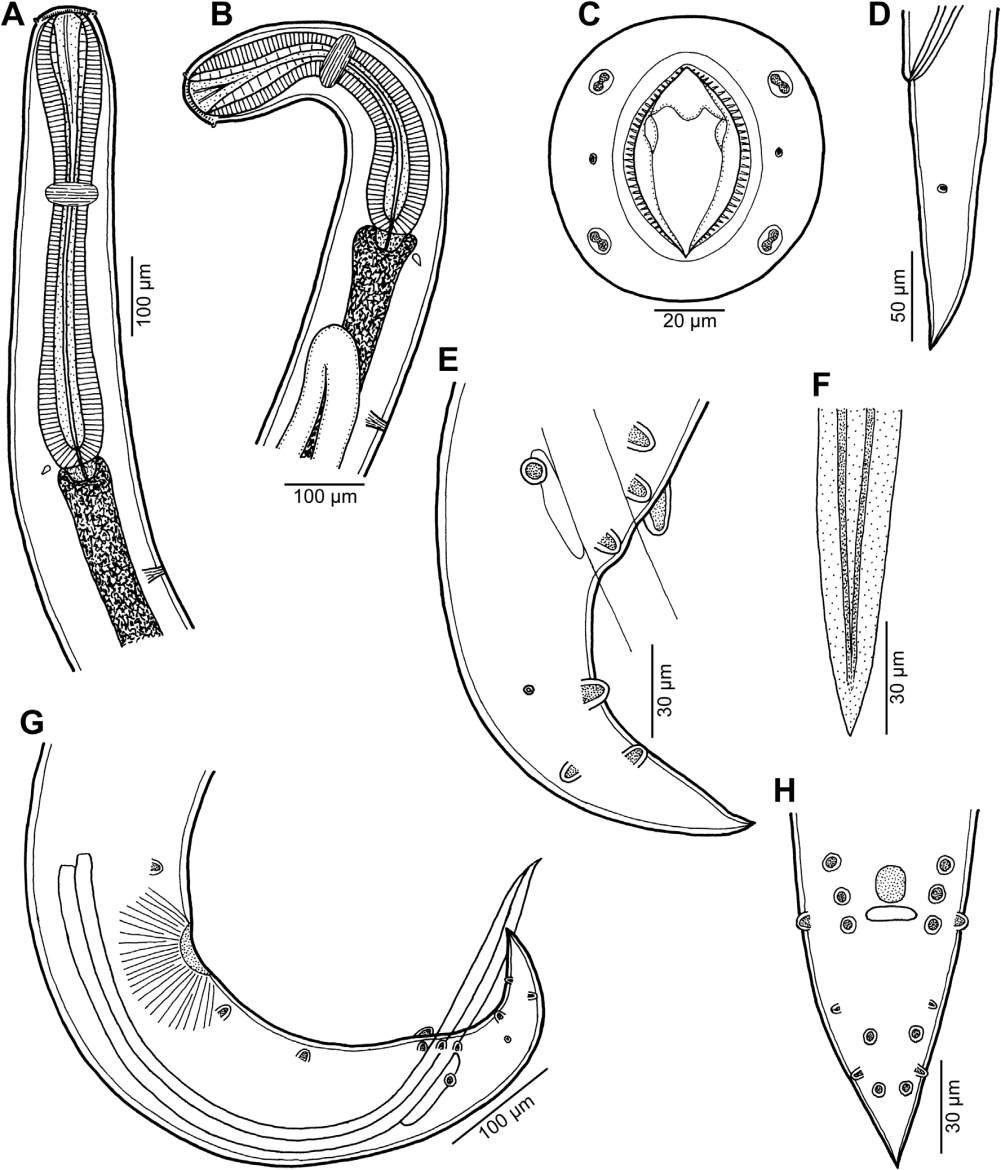
*Cucullanus acutospiculatus* n. sp. from *Caesio cuning*. (A, B) Anterior end of male, lateral views (two different specimens); (C) cephalic end, apical view; (D) tail of juvenile female, lateral view; (E) tail of male, lateral view; (F) distal end of spicule, lateral view; (G) posterior end of male, lateral view; (H) tail of male, ventral view.

Type host: Redbelly yellowtail fusilier *Caesio cuning* (Bloch) (Caesionidae, Perciformes).

Site of infection: Intestine.

Type locality: Fish market, Nouméa, New Caledonia (collected 5 and 16 December 2008) (JNC2815, JNC2816, JNC2851, JNC2852).

Prevalence, intensity and details about fish: 4 fish infected/8 fish examined; 1–2 nematodes; the examined fish were 200–274 mm in fork length and 175–511 g in weight. The photograph of one of the examined fish has been deposited in Wikimedia as https://commons.wikimedia.org/wiki/File:Caesio_cuning.jpg).

Deposition of type specimens: Helminthological Collection, Institute of Parasitology, Biology Centre of the Czech Academy of Sciences, České Budějovice, Czech Republic (holotype and 1 paratype in vial and allotype and 2 paratypes mounted on SEM stubs, IPCAS N–1218).

Etymology: The specific name *acutospiculatus* (= with pointed spicules) is the Latin adjective, derived from the words *acutus* (= pointed) and *spiculum* (= spicule), and it relates to the characteristic feature of this species, i.e. the presence of spicules with pointed distal tips.

#### Description

**Figure 5:**
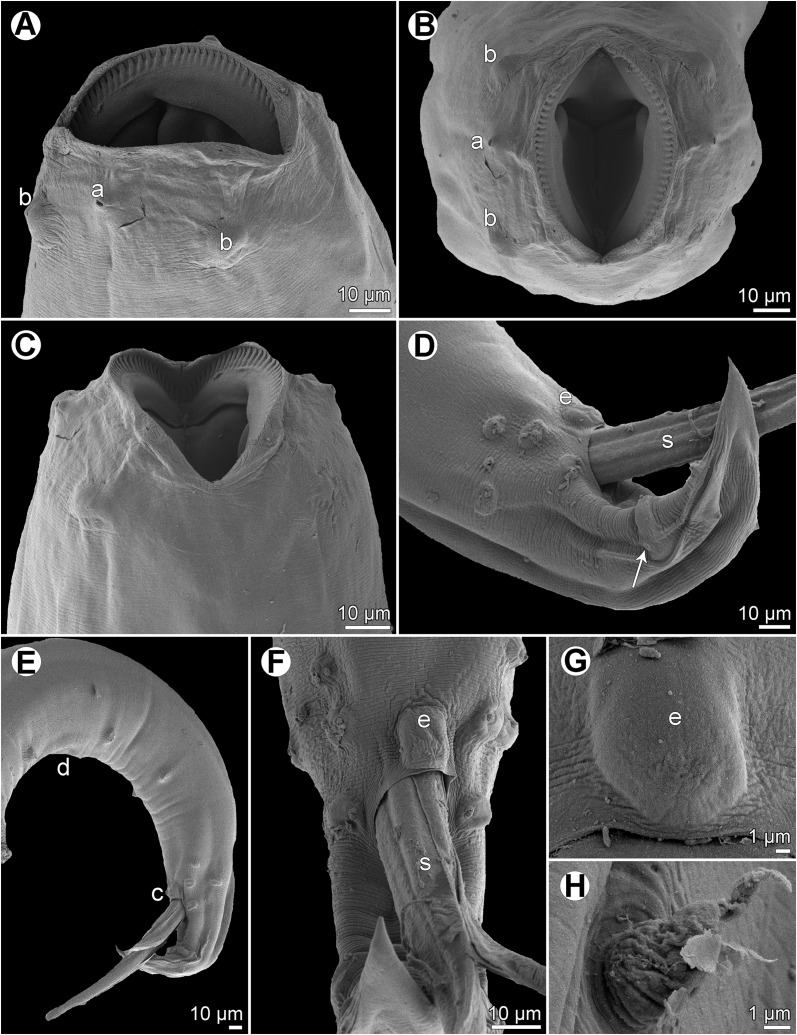
*Cucullanus acutospiculatus* n. sp. from *Caesio cuning*, scanning electron micrographs of male. (A)–(C) Cephalic end, lateral, apical and dorsoventral views, respectively; (D) tail, lateral view (arrow indicates phasmid); (E) posterior end of body, lateral view; (F) cloacal region, ventral view; (G) median precloacal elevation, ventral view; (H) deirid. (a) Amphid; (b) cephalic papilla; (c) cloaca; (d) ventral sucker; (e) median precloacal elevation; (s) spicule.

*General*: Small nematodes with whitish, elongate body. Lateral alae absent. Oral aperture dorsoventrally elongate, surrounded by raised narrow membranous ala (collarette) supported by row of c. 70 minute basal teeth (Figs. 4C, 5A–5C). Four submedian cephalic double papillae and pair of lateral amphids present (Figs. 4C, 5A–5C). Oesophagus muscular, expanded at anterior end to form elongate pseudobuccal capsule (oesophastome); posterior part of oesophagus also expanded, as wide as oesophastome or slightly narrower in lateral view (Fig. 4A, 4B). Oesophagus opens into intestine through valve. Nerve ring encircles oesophagus at 39–45% of oesophagus length. Deirids small, pointed, situated near level of oesophago-intestinal junction (Figs. 4A, 4B, 5H). Postdeirids not found. Excretory pore situated some distance posterior to end of oesophagus (Figs. 4A, 4B). Tail of both sexes conical, sharply pointed at tip.

**Figure 6:**
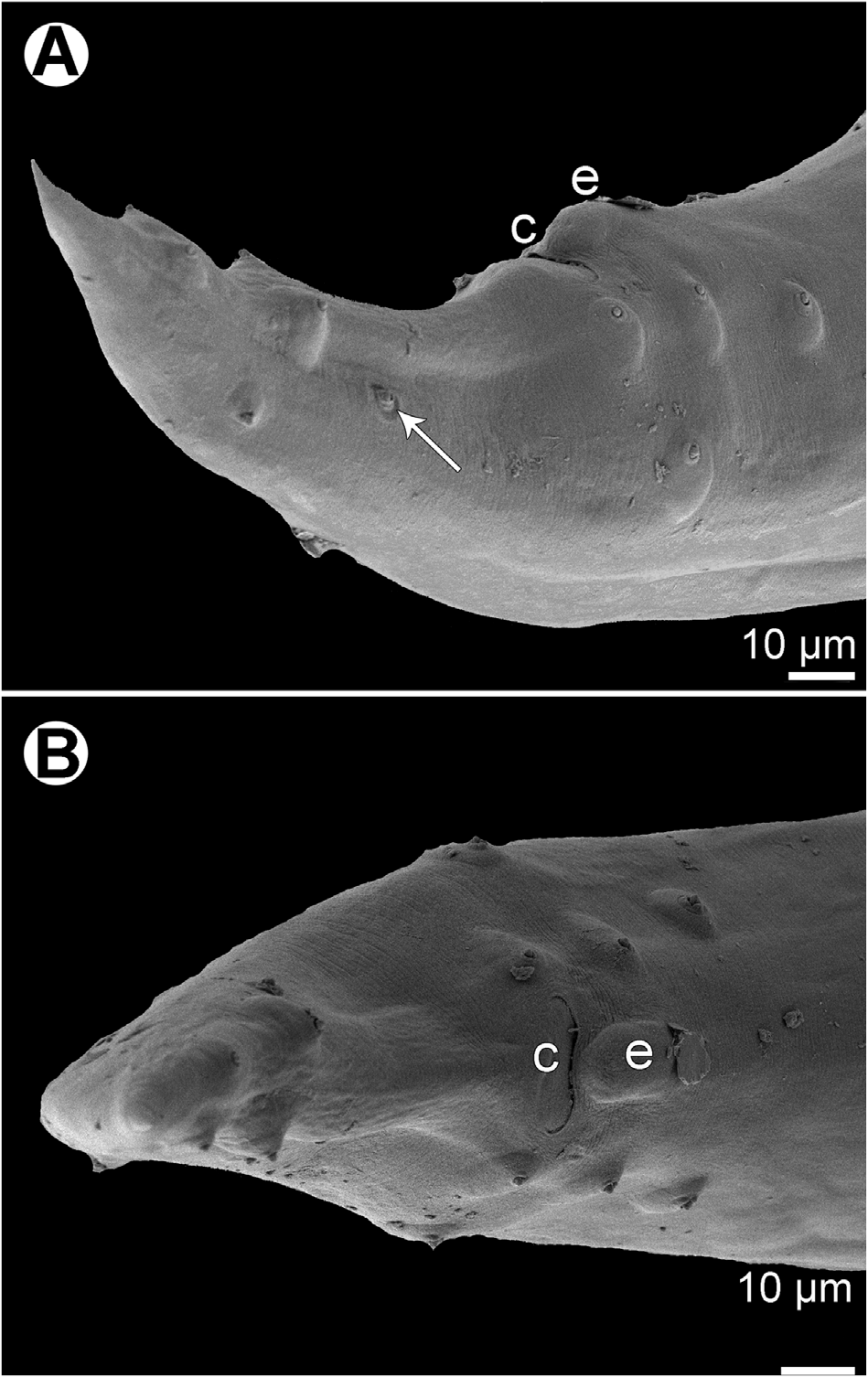
*Cucullanus acutospiculatus* n. sp. from *Caesio cuning*, scanning electron micrographs of male tail (another specimen). (A) Lateral view (arrow indicates phasmid); (B) ventral view. (c) Cloaca; (e) median precloacal elevation.

*Male* (3 specimens; measurements of holotype in parentheses. Measurements of an additional juvenile specimen [spicules weakly sclerotized, ventral sucker indistinct] in brackets): Length of body 4.77–7.49 (7.49) [3.15] mm, maximum width 177–245 (231) [150]; width at level of oesophastome 109–122 (122) [82], at middle of oesophagus 122–177 (177) [109]. Length of entire oesophagus 517–680 (680) [503], representing 9–13% (9%) [16%] of whole body length; length of oesophastome 177–204 (204) [150], its width 95–109 (109) [82]; minimum width of oesophagus 41–54 (54) [41]; maximum width of posterior part of oesophagus 82–95 (95) [68] (Figs. 4A, 4B). Distance of nerve ring from anterior extremity 231–272 (272) [218], representing 39–45% (40%) [43%] of oesophagus length. Deirids and excretory pore 530–721 (721) [530] and 707–952 (952) [666], respectively, from anterior end of body (Figs. 4A, 4B, 5H). Posterior end of body curved ventrally. Ventral sucker present (Figs. 4G, 5E). Cloacal region not elevated. Small median, oval cuticular elevation without papillae on its surface, present anterior to cloacal opening (Figs. 4E, 4G, 4H, 5D–5G, 6A, 6B). Spicules alate, equal, 653–775 (775) [490] long, representing 10–14% (10%) [16%] of body length (Figs. 4G, 5E); distal tips of spicules sharply pointed (Fig. 4F). Gubernaculum 63–69 (69) [60] long, rod-like in lateral view and Y-shaped in ventral view (Figs. 4E, 4G). Caudal papillae 10 pairs: 5 pairs of subventral preanal papillae, 2 pairs of adanal papillae (1 subventral and 1 lateral) and 3 pairs of postanal papillae (2 subventral and 1 dorsolateral); first subventral pair of postanals somewhat posterior to level of cloacal aperture, second and third subventral pairs of postanals somewhat anterior to tail tip; papillae of dorsolateral postanal pair slightly anterior to level of last pair of subventrals (Figs. 4E, 4G, 4H, 5D–5F, 6A, 6B). Pair of small lateral phasmids slightly anterior to level of second subventral pair of postanal papillae (Figs. 4E, 4G, 4H, 5D, 6A). Length of tail 156–204 (204) [136] (Figs. 4E, 4G, 4H, 5D, 5E, 6A).

*Female* (1 nongravid specimen, allotype): Length of body 3.31 mm, maximum width 109; width at level of oesophastome 109, at middle of oesophagus 109. Length of entire oesophagus 449, representing 14% of whole body length; length of oesophastome 122, its width 82; minimum width of oesophagus 27; maximum width of posterior part of oesophagus 54. Distance of nerve ring from anterior extremity 177, representing 39% of oesophageal length. Deirids and excretory pore 476 and 666, respectively, from anterior end of body. Vulva postequatorial, 2.08 mm from anterior extremity, at 63% of body length; vulval lips elevated. Vagina directed anteriorly from vulva. Uteri opposed, empty. Tail 150 long, with pointed tip; lateral phasmids situated approximately at its middle (Fig. 4D).

#### Remarks

To date, the only species of *Cucullanus* reported from a fish host belonging to the perciform family Caesionidae is *C*. *bourdini*. This species was originally described from three species of lutjanid fishes off New Caledonia [23] and, subsequently, *C*. *bourdini* was reported from three host species belonging to the Lutjanidae, Holocentridae and Balistidae from off French Polynesia [8]. Recently, *C*. *bourdini* was reported from the tetraodontid fish *Arothron hispidus* (Linnaeus) from off Palmyra Atoll, East Indo-Pacific [4]. Nevertheless, Moravec and Justine [13], based on LM and SEM examinations of specimens newly collected from two congeneric lutjanid hosts (*Pristipomoides* spp., including the type host *P*. *filamentosus* (Valenciennes)) in New Caledonia, redescribed *C*. *bourdini* and indicated a certain host specificity of this nematode within the sympatric species of the Lutjanidae.

Xu et al. [27], with reference to the descriptions of *C*. *bourdini* by Petter and Le Bel [23] and Moravec and Justine [13], identified nematodes collected from *Caesio xanthonota* Bleeker (Caesionidae, Perciformes) in the Taiwan Strait as *C*. *bourdini*, mentioning that Caesionidae is closely related to the Lutjanidae. Nevertheless, the authors found some morphological differences as compared with the previous descriptions of *C*. *bourdini*, which they considered to be within intraspecific variability. However, although the general morphology and measurements of their nematodes seem to be similar to those of *C*. *bourdini*, the location of the excretory pore and deirids to the oesophago-intestinal junction, considered an important taxonomic feature in *Cucullanus* spp., is very different (deirids and excretory pore conspicuously far posterior to the oesophagus end *versus* deirids at level of the oesophago-intestinal junction or slightly anterior or posterior to it, and excretory pore short distance posterior to deirids in *C*. *bourdini*). Therefore, in our opinion, the specimens from *Caesio xanthonota* represent a new *Cucullanus* species, different from *C*. *bourdini*, for which the name *Cucullanus sinensis* n. sp. is proposed. The new name has been registered in ZooBank as urn:lsid:zoobank.org:act:1A049B06-D1B8-4CC1-9BA0-5104E710A37D.

Generally, the morphology of *C*. *acutospiculatus* n. sp., for example the shape and structure of the oesophagus, the presence of a ventral sucker and a simple median precloacal elevation, the absence of a posterior outgrowth on the anterior cloacal lip, the number and distribution of caudal papillae or the location of the excretory pore and deirids relative to the oesophago-intestinal junction, is similar to that of *C*. *bourdini*; except for the location of deirids and the excretory pore (see above), also to that of *C*. *sinensis* n. sp. However, the males of *C*. *acutospiculatus* n. sp. are much shorter than those of *C*. *bourdini* and *C*. *sinensis* (4.8–7.5 mm *vs.* 9.7–14.0 mm and 10.9–12.1 mm, respectively), their oesophagus is distinctly shorter (517–680 μm *vs.* 1,100–1240 μm and 780–930 μm, respectively) as well as their spicules (653–775 μm *vs.* 740–1000 μm and 900–1070 μm, respectively); the distance of deirids and the excretory pore from the anterior extremity in males is 530–721 μm (*vs.* 830–1,240 μm and 880–980 μm) and 707–952 μm (*vs.* 1510–1560 μm and 1540–1650 μm), respectively. There are c. 70 peribuccal denticles in *C*. *acutospiculatus* n. sp., but c. 90 denticles in each *C*. *bourdini* and *C*. *sinensis*. The nerve ring encircles the oesophagus at 39–43% of its length in *C*. *acutospiculatus* n. sp., whereas at 32–36% and 35–36% in *C*. *bourdini* and *C*. *sinensis*, respectively. In addition, *C*. *acutospiculatus* n. sp. differs from *C*. *bourdini* in the family of the fish host (Caesionidae *vs.* Lutjanidae) and from *C*. *sinensis* in the geographical region (off New Caledonia, South Pacific Ocean *vs.* Taiwan Strait, western North Pacific Ocean) and the host species (*Cae*. *cuning vs. Cae*. *xanthonota*).

### *Cucullanus diagrammae* n. sp. Figures 7, 8

urn:lsid:zoobank.org:act:4DD1D3D7-2127-4FB0-A6C8-C4C2E511CB3D

**Figure 7:**
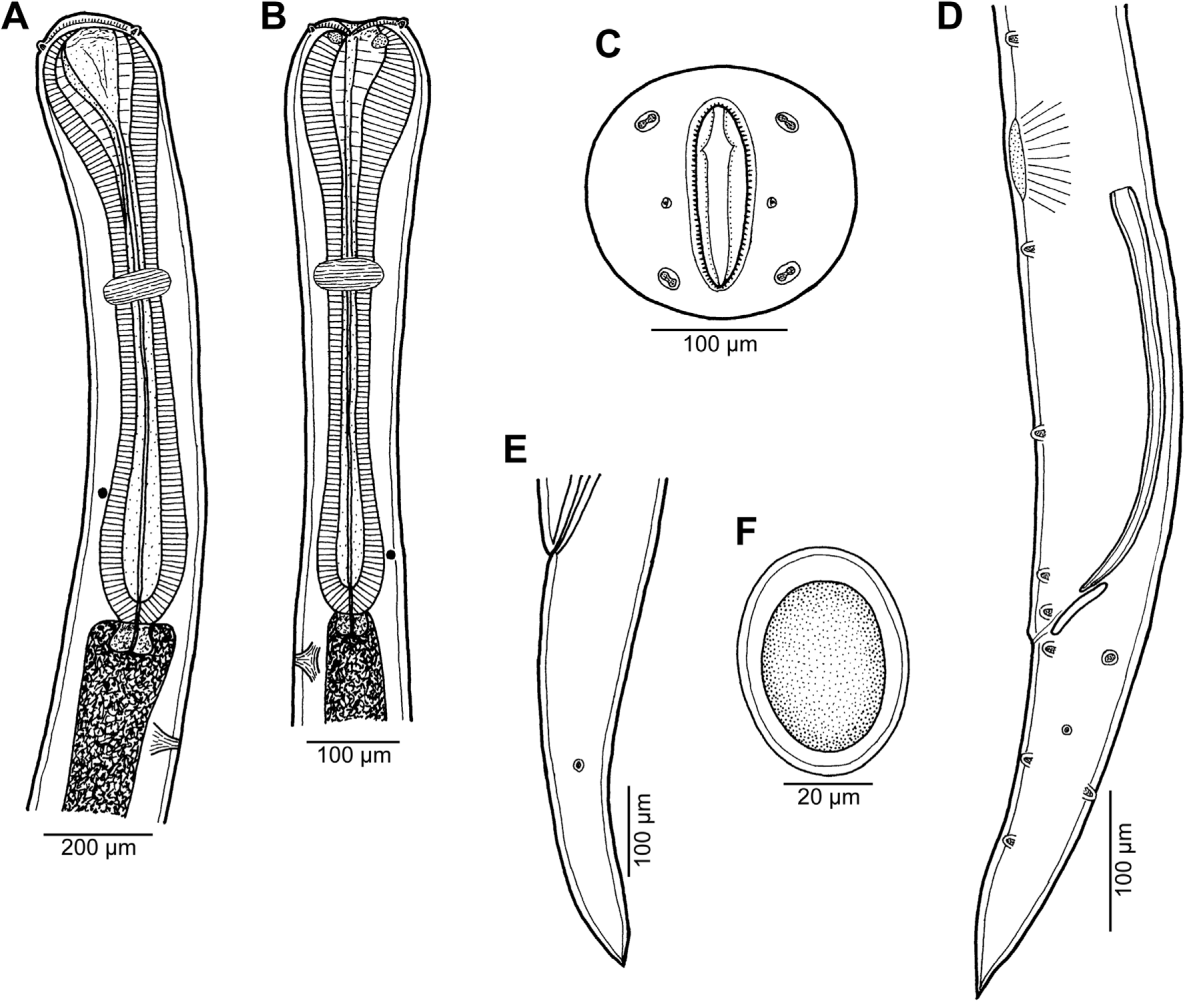
*Cucullanus diagrammae* n. sp. from *Diagramma pictum*. (A, B) Anterior end of male, lateral views (two different specimens); (C) cephalic end, apical view; (D) posterior end of male, lateral view; (E) tail of female, lateral view; (F) egg.

**Figure 8:**
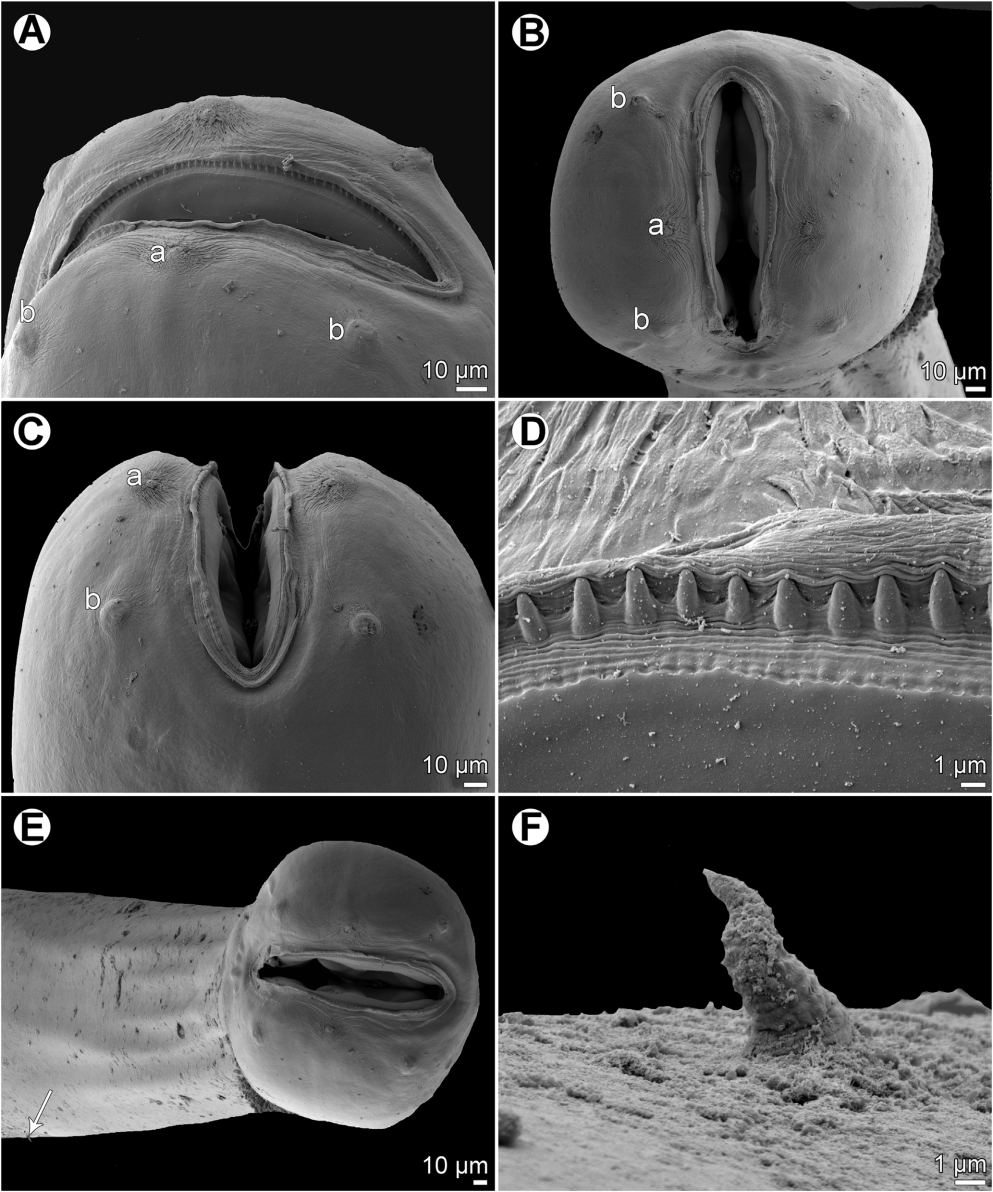
*Cucullanus diagrammae* n. sp. from *Diagramma pictum*, scanning electron micrographs of male. (A, B) Cephalic end, sublateral and apical views, respectively; (C) cephalic end, dorsoventral view; (D) detail of peribuccal teeth; (E) anterior end of body, dorsoventral view (arrow indicates deirid); (F) deirid. (a) Amphid; (b) cephalic papilla.

Type host: Painted sweetlips *Diagramma pictum* (Thunberg) (Haemulidae, Perciformes).

Site of infection: Intestine.

Type locality: Off Ouano, near Ilôt Lebris, New Caledonia, 21°49,622 S, 166°45,353 E (collected 25 October 2007 [fish JNC2342]) and between La Régnière and Récif Crouy, off Nouméa, New Caledonia, 22°20,702 S, 166° 19,295 E, 5 May 2008 [fish JNC2508, JNC2509]).

Prevalence, intensity and details about fish: 3 fish infected/18 fish examined; 1–2 nematodes; the infected fish were 451–530 mm in fork length and 1157–2200 g in weight. Photographs of several examined fish have been deposited in Wikimedia as https://commons.wikimedia.org/wiki/File:Diagramma_pictum_JNC1245.JPG, https://commons.wikimedia.org/wiki/File:Diagramma_pictum_JNC1848.JPG, and https://commons.wikimedia.org/wiki/File:Diagramma_pictum_JNC2512.JPG.

Deposition of type specimens: Helminthological Collection, Institute of Parasitology, Biology Centre of the Czech Academy of Sciences, České Budějovice, Czech Republic (holotype and 1 paratype mounted on SEM stubs and allotype in vial, IPCAS N–1219) and Muséum National d’Histoire Naturelle, Paris, France (1 paratype, MNHN JNC2509).

Etymology: The specific name of this nematode relates to the genitive form of the generic name of the hosts.

#### Description

*General*: Small nematodes with whitish, elongate body. Lateral alae absent. Cephalic end slightly asymmetrical in lateral view (Figs. 7A–7C, 8A, 8B, 8E). Oral aperture dorsoventrally elongate, surrounded by raised narrow membranous ala (collarette) supported by row of c. 100 minute basal teeth (Figs. 7C, 8A–8E). Four submedian cephalic double papillae and pair of lateral amphids present (Figs. 7C, 8A–8C, 8E). Oesophagus muscular, expanded at anterior end to form elongate pseudobuccal capsule (oesophastome) slightly asymmetrical in lateral view; posterior part of oesophagus also expanded, somewhat narrower than oesophastome in lateral view (Figs. 7A, 7B). Oesophagus opens into intestine through large valve. Nerve ring encircles oesophagus approximately at 37–46% of its length. Deirids small, pointed, situated short distance anterior to oesophago-intestinal junction (Figs. 7A, 7B, 8F). Postdeirids not found. Excretory pore situated short distance posterior to end of oesophagus (Figs. 7A, 7B). Tail of both sexes conical, pointed.

*Male* (1 specimen, holotype; measurements of incomplete paratype in parentheses): Length of body 5.39 (of incomplete paratype 10.20) mm, maximum width 163 (354); width at level of oesophastome 163 (245), at middle of oesophagus 109 (204). Length of entire oesophagus 734 (1210), representing 14% of whole body length; length of oesophastome 163 (326), its width 150 (218); minimum width of oesophagus 41 (95); maximum width of posterior part of oesophagus 95 (190). Distance of nerve ring from anterior extremity 340 (503), representing 46 (42)% of oesophageal length. Deirids and excretory pore 666 (1170) and 789 (1469), respectively, from anterior end of body. Posterior end of body somewhat curved ventrally. Ventral sucker present (Fig. 7D). Cloacal region not elevated. Spicules equal, 315 long, representing 5.9% of body length. Gubernaculum 66 long, rod-like in lateral view (Fig. 7D). Caudal papillae 10 pairs: 5 pairs of subventral preanal papillae and 5 pairs of postanal papillae (3 subventral and 2 lateral and dorsolateral); last 2 preanal and 1st postanal pairs of subventrals close to each other; first lateral pair of postanals slightly posterior to level of cloacal aperture; papillae of dorsolateral postanal pair slightly posterior to level of 2nd pair of subventrals (Fig. 7D). Pair of small lateral phasmids somewhat anterior to level of 2nd subventral pair of postanal papillae (Fig. 7D). Length of tail 286 (Fig. 7D).

*Female* (1 gravid specimen, allotype; measurements of 1 gravid paratype specimen in parentheses): Length of body 14.39 (13.79) mm, maximum width 313 (299); width at level of oesophastome 272 (245), at middle of oesophagus 177 (190). Length of entire oesophagus 1.29 (1.27) mm, representing 37 (42)% of whole body length; length of oesophastome 340 (326), its width 218 (204); minimum width of oesophagus 95 (82); maximum width of posterior part of oesophagus 177 (163). Distance of nerve ring from anterior extremity 476 (530), representing 37 (42)% of oesophageal length. Deirids and excretory pore 1.21 (1.21) mm and 1.47 (1.41) mm, respectively, from anterior end of body. Vulva postequatorial, 8.80 (8.65) mm from anterior extremity, at 61 (63)% of body length; vulval lips not elevated. Vagina directed anteriorly from vulva. Uteri opposed. Fully developed eggs elongate-oval, thin-walled, size 66 × 45 (60 × 42), with uncleaved contents (Fig. 7F). Tail 476 (490) long, with pointed tip; small lateral phasmids situated somewhat posterior to its middle (Fig. 7E).

#### Remarks

Smales [24] reported *Cucullanus australiensis* Baylis, 1927 (syn. *C*. *faliexae* Morand et Rigby, 1998 [18]) from three fish hosts, *Caranx ignobilis* (Forsskål) (Carangidae), *Diagramma pictum* (Haemulidae) and *Plectorhinchus schotaf* (Forsskål) (Haemulidae) off the coast of Queensland (Keppel Islands), Australia. However, this species identification was probably incorrect, because *C*. *australiensis* is a parasite of *Gymnothorax* spp. (Muraenidae, Anguilliformes) [2, 8, 15] and its occurrence in perciform fishes is highly improbable. Smales [24] gave no morphological data on these nematodes; the nematodes she mentioned from *D*. *pictum* might be identical to *C*. *diagrammae* n. sp. *Cucullanus australiensis* differs from the new species mainly in having deirids located somewhat posterior to the level of the nerve ring (*vs.* near the end of the oesophagus) and the excretory pore in the region of deirids (*vs.* posterior to the oesophago-intestinal junction), and much longer spicules (990–1200 μm *vs.* 315 μm) [15].

To date, *C*. *diagrammae* n. sp. seems to be the only nominal species of *Cucullanus* parasitizing a host of the perciform family Haemulidae.

### *Cucullanus parapercidis* n. sp. Figures 9–11

urn:lsid:zoobank.org:act:F70AD5B7-71DF-45D2-B07B-CC0559A193CD

**Figure 9:**
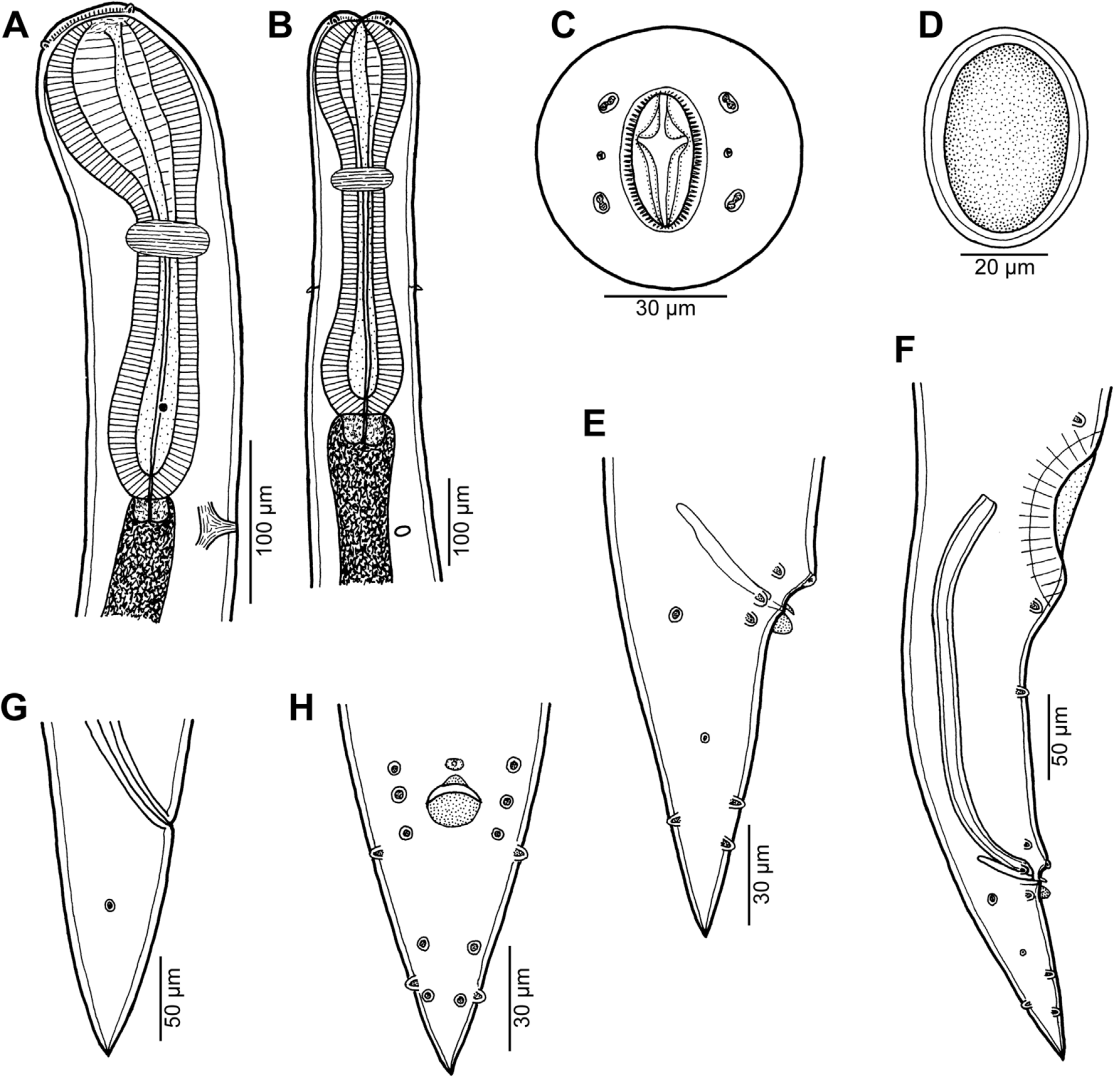
*Cucullanus parapercidis* n. sp. from *Parapercis* spp. (A) Anterior end of male, lateral view; (B) anterior end of female, dorsoventral view; (C) cephalic end, apical view; (D) egg; (E) tail of male, lateral view; (F) posterior end of male, lateral view; (G) tail of female, lateral view; (H) tail of male, ventral view. (A), (C), (E), (F) and (H) from *P*. *xanthozona*; (B), (D) and (G) from *P*. *hexophtalma*.

Type host: Yellowbar sandperch *Parapercis xanthozona* (Bleeker) (Pinguipedidae, Perciformes).

Other host: Speckled sandperch *Parapercis hexophtalma* (Cuvier) (Pinguipedidae, Perciformes).

Site of infection: Intestine.

Type locality: Grande Rade, Nouméa, New Caledonia, 22°13′34″ S, 166°23′41″ E (collected 24 July 2007).

Other locality: Near Récif Snark, off Nouméa, Off New Caledonia (collected 15 May 2008).

Prevalence, intensity and details about fish: *P*. *xanthozona*: 1 fish infected/2 fish examined; 1 nematode; the infected fish (JNC2252) was 165 mm in fork length and 43 g in weight. *P*. *hexophtalma*: 1 fish infected/2 fish examined; 1 nematode; the infected fish (JNC2537) was 210 mm in fork length and 96 g in weight.

Deposition of type specimens: Helminthological Collection, Institute of Parasitology, Biology Centre of the Czech Academy of Sciences, České Budějovice, Czech Republic (holotype and allotype mounted on SEM stubs, IPCAS N–1220).

Etymology: The specific name of this nematode relates to the genitive form of the generic name of the hosts.

#### Description

**Figure 10:**
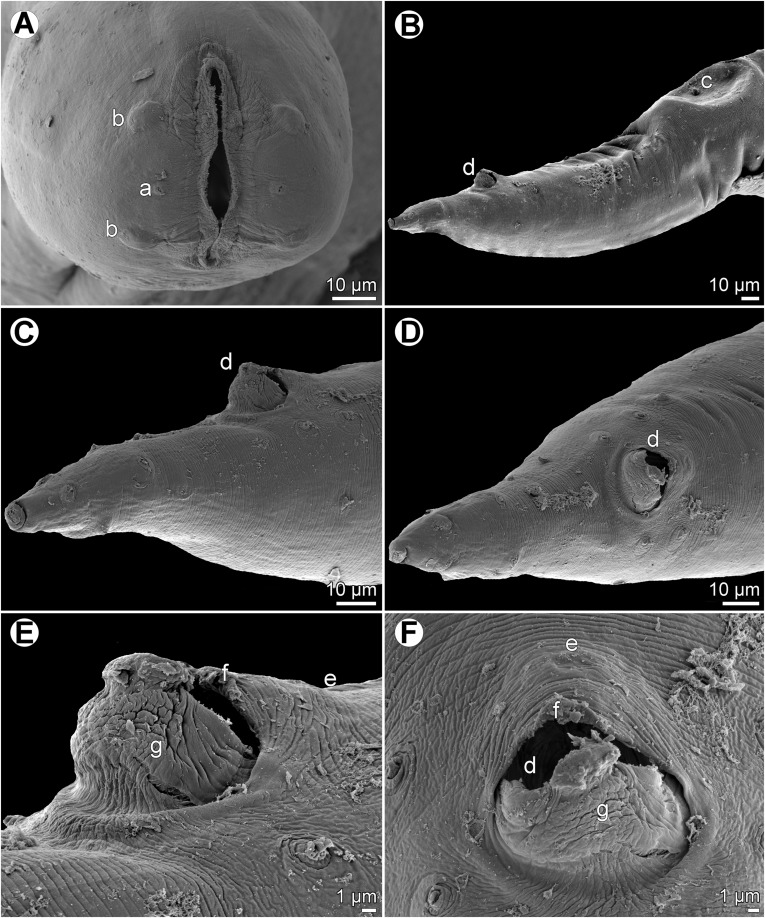
*Cucullanus parapercidis* n. sp., scanning electron micrographs of male from *Parapercis xanthozona*. (A) Cephalic end, apical view; (B) posterior end of body, lateral view; (C, D) tail, lateral and ventral views, respectively; (E, F) region of cloaca, lateral and ventral views, respectively. (a) Amphid; (b) cephalic papilla; (c) ventral sucker; (d) cloacal aperture; (e) precloacal median elevation; (f) outgrowth of anterior cloacal lip; (g) posterior cloacal lip with apical outgrowth.

**Figure 11:**
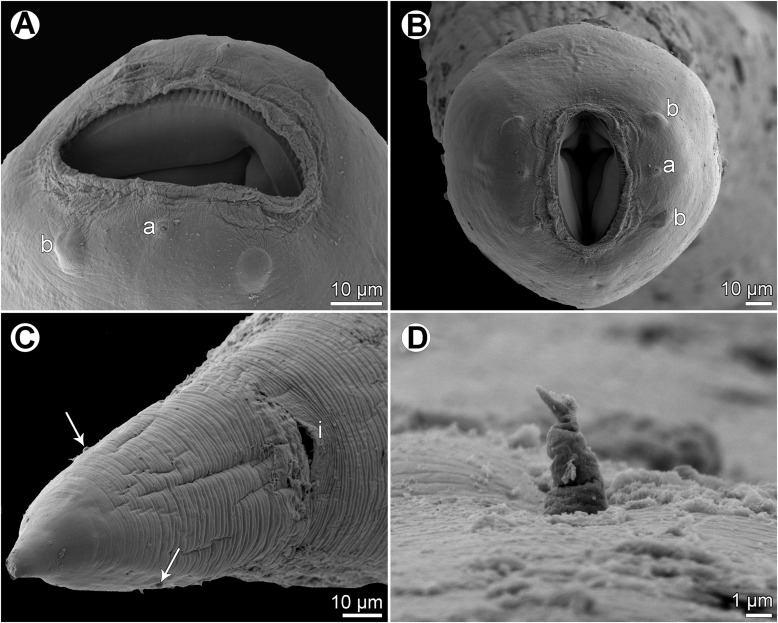
*Cucullanus parapercidis* n. sp., scanning electron micrographs of gravid female from *Parapercis hexophtalma*. (A, B) Cephalic end, sublateral and apical views, respectively; (C) tail, ventral view (arrows indicate phasmids); (D) deirid. (a) Amphid; (b) cephalic papilla; (i) anus.

*General*: Small nematodes with whitish, elongate body. Lateral alae absent. Cephalic end slightly asymmetrical in lateral view (Fig. 9A). Oral aperture dorsoventrally elongate, surrounded by raised narrow membranous ala (collarette) supported by row of many minute basal teeth (Figs. 9C, 10A, 11A, 11B). Four submedian cephalic double papillae and pair of lateral amphids present (Figs. 9C, 10A, 11A, 11B). Oesophagus muscular, expanded at anterior end to form large pseudobuccal capsule (oesophastome) somewhat asymmetrical in lateral view; posterior part of oesophagus also expanded, somewhat narrower as oesophastome (Figs. 9A, 9B). Oesophagus opens into intestine through large valve. Nerve ring encircles oesophagus at 39–48% of its length. Deirids small, pointed, situated approximately at mid-way between nerve ring and oesophago-intestinal junction or somewhat posterior to it (Figs. 9A, 9B, 11D). Postdeirids not found. Excretory pore situated short distance posterior to end of oesophagus (Figs. 9A, 9B). Tail of both sexes conical, pointed.

*Male* (1 specimen from *P*. *xanthozona*, holotype): Length of body 1.48 mm, maximum width 122; width at level of oesophastome 99, at middle of oesophagus 99. Length of entire oesophagus 326, representing 22% of whole body length; length of oesophastome 135, its width 93; minimum width of oesophagus 33; maximum width of posterior part of oesophagus 54. Distance of nerve ring from anterior extremity 156, representing 48% of oesophageal length. Deirids and excretory pore 237 and 340, respectively, from anterior end of body. Posterior end of body slightly curved ventrally. Ventral sucker present (Figs. 9F, 10B). Cloacal region somewhat elevated. Anterior cloacal lip with small median transversely oval cuticular outgrowth not covering cloacal aperture. Small median, transversely oval cuticular elevation without papillae present anterior to cloacal outgrowth (Figs. 9E, 9F, 9H, 10B–10F); posterior cloacal lip large, bearing small transversely oval elevation (Figs. 9E, 9F, 9H, 10B–10F). Spicules equal, 282 long, representing 19% of body length. Gubernaculum well sclerotized, 51 long, rod-like in lateral view (Figs. 9E, 9F). Caudal papillae 10 pairs: 5 pairs of subventral preanal papillae and 5 pairs of postanal papillae (3 subventral and 2 lateral and dorsolateral); second pair of preanals just posterior to ventral sucker; last 2 pairs of preanals and first pair of subventral postanals close to each other; first lateral pair of postanals somewhat posterior to level of cloacal aperture; papillae of dorsolateral postanal pair slightly anterior to level of last pair of subventrals (Figs. 9E, 9F, 9H, 10B–10D). Pair of small lateral phasmids somewhat anterior to level of second subventral pair of postanal papillae (Figs. 9E, 9F, 9H, 10C, 10D). Length of tail 123 (Figs. 9E, 9F, 9H, 10B–10D).

*Female* (1 gravid specimen from *P*. *hexophtalma*, allotype): Length of body 4.62 mm, maximum width 204; width at level of oesophastome 136, at middle of oesophagus 109. Length of entire oesophagus 517, representing 11% of whole body length; length of oesophastome 163, its width 122; minimum width of oesophagus 54; maximum width of posterior part of oesophagus 109 (Fig. 9B). Distance of nerve ring from anterior extremity 204, representing 39% of oesophageal length. Deirids and excretory pore 476 and 666, respectively, from anterior end of body (Fig. 9B). Vulva postequatorial, 2.58 mm from anterior extremity, at 56% of body length; vulval lips not elevated. Vagina directed anteriorly from vulva. Uterus contains many eggs; eggs oval, thin-walled, size 69–72 × 45–48, with uncleaved contents (Fig. 9D). Tail 153 long, with pointed tip; small lateral phasmids situated approximately at its middle (Figs. 9G, 11C).

#### Remarks

To date, only two nominal species of *Cucullanus* have been described from fishes belonging to the perciform family Pinguipedidae: *C*. *carioca* Vicente et Fernandes, 1973 from *Pinguipes brasilianus* Cuvier and *C*. *pseudopercis* Pereira, Vieira et Luque in Viera et al., 2015 from *Pseudopercis semifasciata* (Cuvier), both from off the Atlantic coast of Brazil [25, 26]. The new species can be easily distinguished from *C*. *carioca* and *C*. *pseudopercis* by conspicuously shorter spicules (282 μm *vs.* 0.91–1.05 mm and 1.10–1.50 mm, respectively), smaller body lengths (male 1.48 mm, female 4.62 mm *vs.* males 4.47–7.71 mm and 8.9–12.1 mm, females 5.88 mm and 10.8–12.7 mm, respectively) and by a different distribution of caudal papillae. Moreover, the deirids of *C*. *pseudopercis* are located posteriorly (*vs.* anteriorly) to the oesophago-intestinal junction and its posterior cloacal lip is not conspicuously larger and higher than the anterior cloacal lip (*vs.* posterior cloacal lip much larger and higher than the anterior cloacal lip). In addition to morphological features, *C*. *parapercidis* n. sp. also differs from *C*. *carioca* and *C*. *pseudopercis* in the host genus (*Parapercis* Bleeker *vs. Pinguipes* Cuvier and *Pseudopercis* Miranda Ribeiro) and a distant geographical region (Pacific Ocean *vs.* Atlantic Ocean).

### *Cucullanus petterae* n. sp. Figures 12–14

urn:lsid:zoobank.org:act:35918549-6AC7-4DE0-BEFB-F4220AB9C3FA

**Figure 12:**
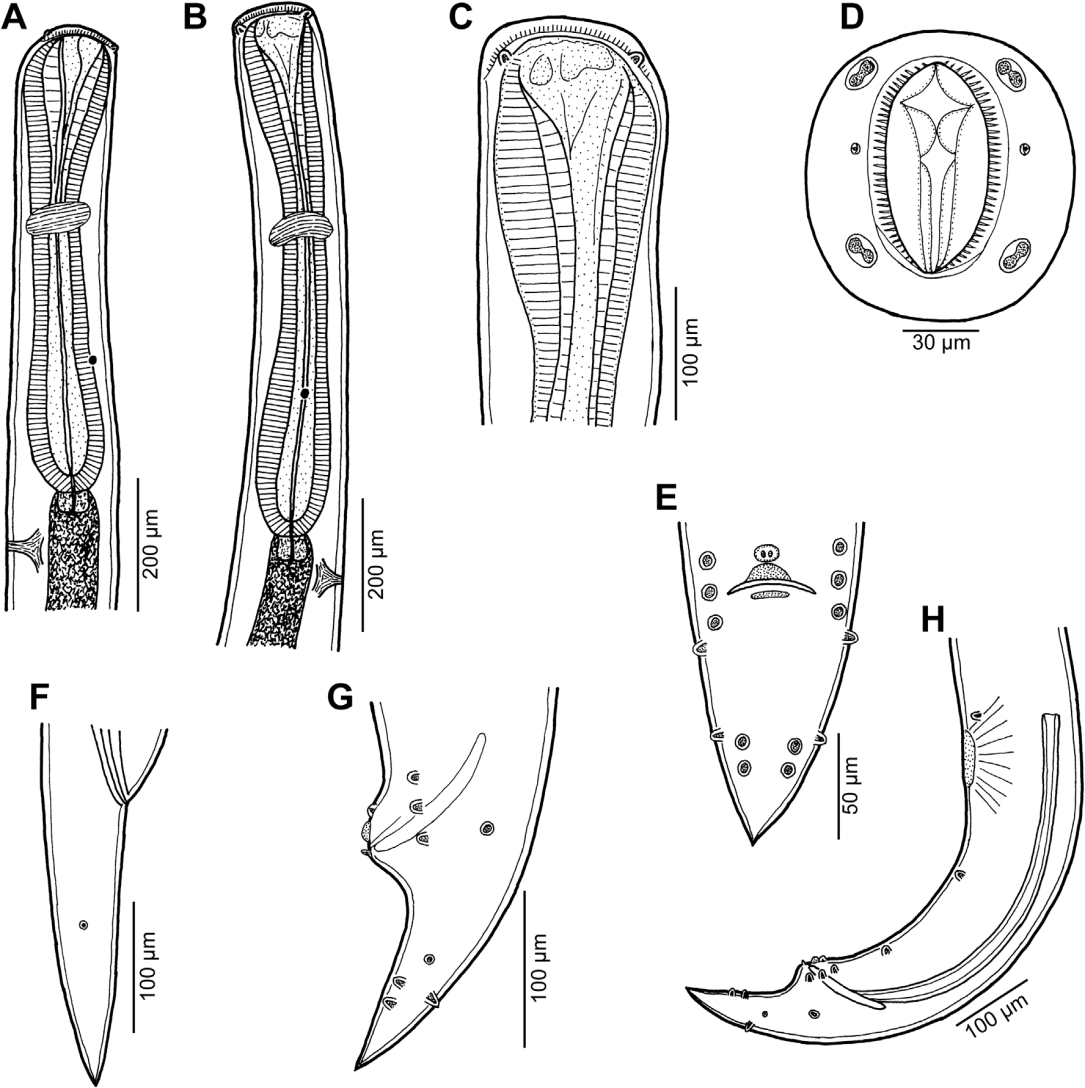
*Cucullanus petterae* n. sp. from *Epinephelus* spp. (A) Anterior end of male, lateral view; (B) anterior end of female, lateral view; (C) pseudobuccal capsule of female, lateral view; (D) cephalic end of female, apical view; (E) tail of male, ventral view; (F) tail of female, lateral view; (G) tail of male, lateral view; (H) posterior end of male, lateral view. (A), (E), (G) and (H) from *Epinephelus merra*; (B), (C), (D) and (F) from *Epinephelus fasciatus*.

Type host: Honeycomb grouper *Epinephelus merra* Bloch (Serranidae, Perciformes).

Other host: Blacktip grouper *Epinephelus fasciatus* (Forsskål) (Serranidae, Perciformes).

Site of infection: Intestine.

Type locality: Côte Blanche, off Nouméa, New Caledonia (collected 18 and 25 November 2005).

Prevalence, intensity and details about fish: *E*. *merra*: 1 fish infected/18 fish examined [5]; 1 nematode; the infected fish (JNC1649) was 170 mm in fork length and 66 g in weight. *E*. *fasciatus*: 1/61 fish examined [5]; 1 nematode; the infected fish (JNC1658) was 180 mm in fork length and 88 g in weight.

Deposition of type specimens: Helminthological Collection, Institute of Parasitology, Biology Centre of the Czech Academy of Sciences, České Budějovice, Czech Republic (holotype and allotype mounted on SEM stubs, IPCAS N–1221).

Etymology: This nematode species is named for the late, eminent French nematodologist Annie J. Petter (1932–2017), who contributed greatly to the knowledge of fish nematodes.

#### Description

**Figure 13:**
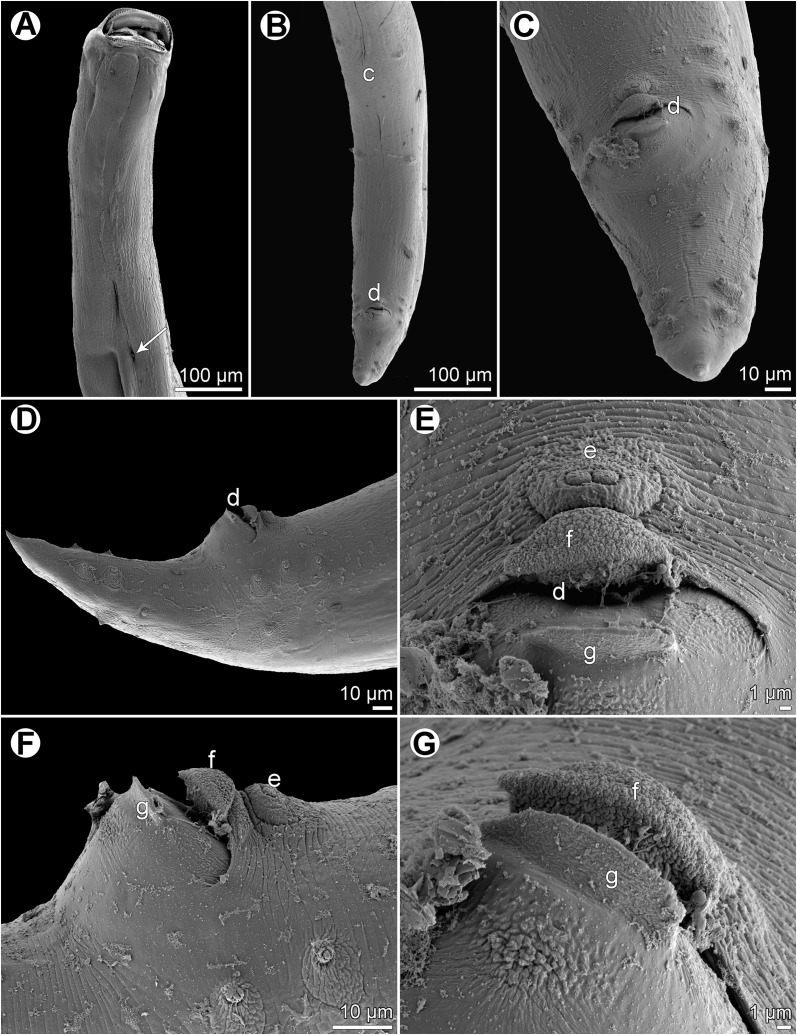
*Cucullanus petterae* n. sp. n., scanning electron micrographs of male from *Epinephelus merra*. (A) Anterior end, lateral view (arrow indicates deirid); (B) posterior end, ventral view; (C) tail, ventral view; (D) tail, lateral view; (E, F) region of cloaca, ventral and lateral views, respectively; (G) cloaca, sublateral view. (c) Ventral sucker; (d) cloacal aperture; (e) precloacal median elevation containing two small papillae; (f) outgrowth of anterior cloacal lip; (g) outgrowth of posterior cloacal lip.

**Figure 14:**
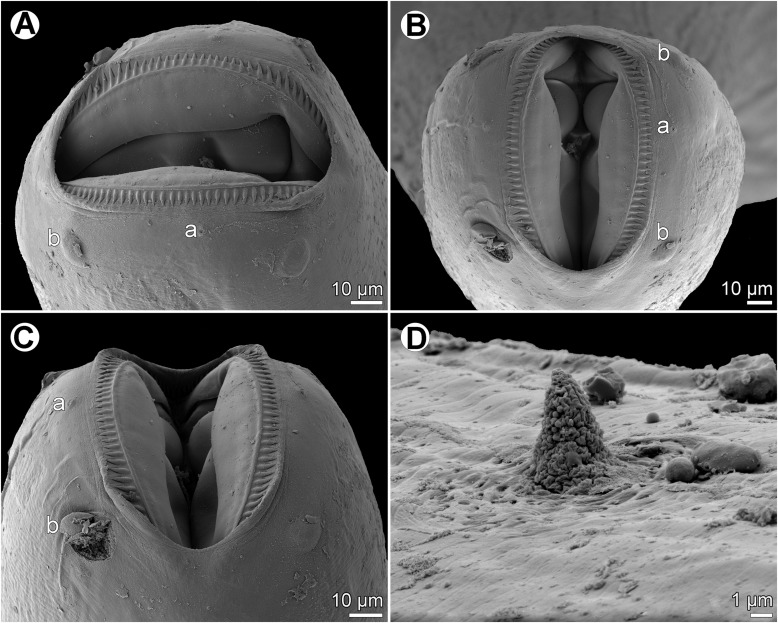
*Cucullanus petterae* n. sp., scanning electron micrographs of nongravid female from *Epinephelus fasciatus*. (A, B) Cephalic end, sublateral and apical views, respectively; (C) cephalic end, dorsoventral view; (D) deirid. (a) Amphid; (b) cephalic papilla.

*General*: Small nematodes with whitish, elongate body. Lateral alae absent. Cephalic end slightly asymmetrical in lateral view (Figs. 12A–12D, 14A, 14B). Oral aperture dorsoventrally elongate, surrounded by raised narrow membranous ala (collarette) supported by row of c. 80 minute basal teeth (Figs. 12C, 12D, 14A–14C). Four submedian cephalic double papillae and pair of lateral amphids present (Figs. 12C, 12D, 14A–14C). Oesophagus muscular, expanded at anterior end to form elongate pseudobuccal capsule (oesophastome) slightly asymmetrical in lateral view; posterior part of oesophagus also expanded, somewhat narrower or approximately as wide as oesophastome in lateral view (Figs. 12A–12C). Oesophagus opens into intestine through large valve. Nerve ring encircles oesophagus at 39– 41% of its length. Deirids small, pointed, situated approximately at mid-way between nerve ring and oesophago-intestinal junction or somewhat posterior to it (Figs. 12A, 12B, 13A, 14D). Postdeirids not found. Excretory pore situated short distance posterior to end of oesophagus (Figs. 12A, 12B). Tail of both sexes conical, pointed.

*Male* (1 specimen from *E*. *merra*, holotype): Length of body 4.201 mm, maximum width 190; width at level of oesophastome 150, at middle of oesophagus 136. Length of entire oesophagus 762, representing 19% of whole body length; length of oesophastome 204, its width 136; minimum width of oesophagus 82; maximum width of posterior part of oesophagus 122. Distance of nerve ring from anterior extremity 299, representing 39% of oesophageal length. Deirids and excretory pore 544 and 843, respectively, from anterior end of body. Posterior end of body curved ventrally. Ventral sucker present (Figs. 12H, 13B). Cloacal region somewhat elevated. Anterior cloacal lip with large median transversely oval cuticular outgrowth not covering cloacal aperture. Small median, transversely oval cuticular elevation bearing two small papillae present just anterior to cloacal outgrowth (Figs. 12E, 12G, 13D–13G); posterior cloacal lip with rugged surface and bearing large transversely oval elevation (Figs. 12E, 12G, 13E–13G). Spicules equal, 501 long, representing 12.5% of body length. Gubernaculum well sclerotized, 117 long, rod-like in lateral view and Y-shaped in ventral view (Fig. 12G, 12H). Caudal papillae 10 pairs: 4 pairs of subventral preanal papillae, 1 pair of subventral adanal papillae and 5 pairs of postanal papillae (3 subventral and 2 lateral and dorsolateral); second pair of preanals rather far posterior to ventral sucker; last preanal, adanal and first postanal pairs of subventrals close to each other; first lateral pair of postanals somewhat posterior to level of cloacal aperture; papillae of dorsolateral postanal pair slightly anterior to level of last pair of subventrals (Figs. 12E, 12G, 12H, 13B–13D). Pair of small lateral phasmids somewhat anterior to level of second subventral pair of postanal papillae (Figs. 12E, 12G, 12H, 13C, 13D). Length of tail 150 (Figs. 12E, 12G, 12H, 13B–13D).

*Female* (1 nongravid specimen from *E*. *fasciatus*, allotype): Length of body 6.42 mm, maximum width 218; width at level of oesophastome 150, at middle of oesophagus 136. Length of entire oesophagus 870, representing 14% of whole body length; length of oesophastome 218, its width 150; minimum width of oesophagus 68; maximum width of posterior part of oesophagus 136 (Figs. 12B, 12C). Distance of nerve ring from anterior extremity 354, representing 41% of oesophageal length. Deirids and excretory pore 645 and 966, respectively, from anterior end of body (Fig. 12B). Vulva postequatorial, 3.67 mm from anterior extremity, at 57% of body length; vulval lips not elevated. Vagina directed anteriorly from vulva. Uterus empty. Tail 245 long, with pointed tip; small lateral phasmids situated approximately at its middle (Fig. 12F).

#### Remarks

Of the three nominal species of *Cucullanus* parasitizing serranid fishes, i.e. *C*. *epinepheli*, *C*. *mycteropercae* and *C*. *variolae* n. sp. (see above), only *C*. *epinepheli* possesses a large cuticular outgrowth on the anterior cloacal lip and two papillae on the median precloacal elevation as the new species. However, in contrast to *C*. *petterae* n. sp., the anterior cloacal outgrowth of *C*. *epinepheli* extends posteriorly to cover the cloacal aperture (*vs.* cloacal outgrowth not covering the cloaca) and there is no elevation on the posterior cloacal lip (*vs.* posterior cloacal lip bears a conspicuous broad elevation). Moreover, *C*. *epinepheli* also differs in the presence (*vs.* absence) of cervical alae, longer spicules (748–789 μm *vs.* 501 μm) and the body length of males (7.9–9.0 mm *vs.* 4.01 mm); the anterior ends of its withdrawn spicules are posterior (*vs.* anterior) to the precloacal sucker. *Cucullanus mycteropercae* also differs from the new species in having only a small outgrowth on the anterior cloacal lip, absence of a cuticular elevation on the posterior cloacal lip and the presence of postdeirids, whereas *C*. *variolae* n. sp. in the absence of an outgrowth on the anterior cloacal lip, the absence of an elevation on the posterior cloacal lip and the situation of the excretory pore anterior to the level of the oesophago-intestinal junction.

### *Cucullanus bioccai* Orecchia et Paggi, 1987 Figure 15

Host: Flathead grey mullet *Mugil cephalus* Linnaeus (Mugilidae, Mugiliformes).

**Figure 15:**
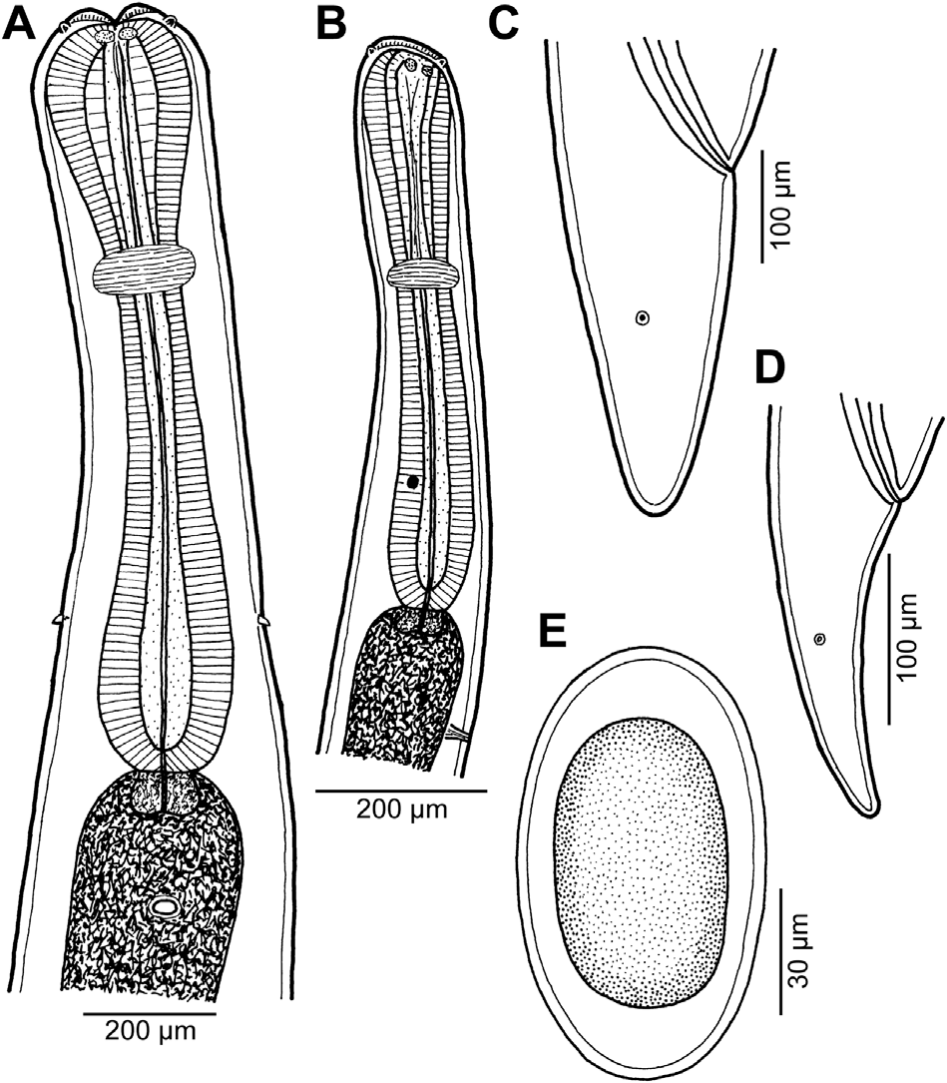
*Cucullanus bioccai* Orecchia et Paggi, 1987 from *Mugil cephalus*, female. (A) Anterior end of gravid specimen, ventral view; (B) anterior end of nongravid specimen, lateral view; (C, D) tail of gravid and nongravid specimens, respectively, lateral views; (E) egg.

Site of infection: Digestive tract.

Locality: Anse Vata, New Caledonia (collected 16 June 2007) (JNC2180).

Prevalence, intensity and details about fish: 1 fish infected/5 fish examined; 2 nematodes; the infected fish (JNC2180) was 465 mm in fork length and 1,730 g in weight.

Deposition of voucher specimens: Muséum National d’Histoire Naturelle, Paris, France (2 specimens, MNHN JNC2180).

#### Description

*Female* (1 gravid specimen; measurements of 1 nongravid specimen in parentheses): Medium-sized nematode with whitish, elongate body. Body length 15.01 (5.69) mm, maximum width 530 (190); width at level of oesophastome 258 (136), at mid-length of oesophagus 272 (122). Lateral alae absent. Oral aperture surrounded by raised narrow membranous ala (collarette) supported by row of numerous minute basal teeth. Four submedian cephalic papillae and pair of lateral amphids present. Oesophagus muscular, 1238 (707) long, representing 8 (12)% of body length, expanded at anterior end to form pseudobuccal capsule (oesophastome) 258 (163) long and 231 (122) wide; posterior part of oesophagus also expanded, 231 (95) wide; minimum width of oesophagus 136 (68) (Figs. 15A, 15B). Nerve ring encircles oesophagus at 35 (38)% of oesophagus length, at 435 (272) from anterior extremity. Deirids situated short distance anterior to level of oesophagointestinal junction, at 993 (571) from anterior end of body (Figs. 15A, 15B). Excretory pore situated some distance posterior to end of oesophagus, 1673 (966) from anterior extremity (Figs. 15A, 15B). Tail of both sexes conical, sharply pointed at tip. Vulva postequatorial, 9.0 (3.59) mm from anterior extremity, at 60 (63)% of body length; vulval lips elevated. Vagina directed anteriorly from vulva. Uterus filled with many oval eggs with unembryonated contain (uterus empty in nongravid specimen); size of eggs 96–111 × 63–72 (Fig. 15E). Tail conical, 340 (204) long, with almost rounded tip; lateral phasmids situated near its middle (Figs. 15C, 15D).

#### Remarks

Since conspecific males are absent, the species identification of these nematodes is based solely on female morphology. Both the morphology and measurements of these nematodes are very close to those of *C*. *bioccai*, as described from the same host species, *M*. *cephalus*, in Italy [20] and are, therefore, considered to belong to this species. Although the deirids of *C*. *bioccai* were illustrated as being located at the level of the oesophagus posterior end, these are largely situated anteriorly to the end of the oesophagus according to the original species description [20].

This is the first record of *C*. *bioccai* from off New Caledonia and in the region of the Pacific Ocean. The presence of this parasite, originally described in Italy, in New Caledonian waters is not surprising, because its host, *M*. *cephalus*, is cosmopolitan in coastal waters of the tropical, subtropical and temperate zones of all seas [3]. It is known that the distribution area of a parasite often coincides with that of its definitive host. Similar cases when the same parasite was recorded from the same host species both in the Atlantic and Pacific regions have recently been reported by Barton et al. [1] and Moravec and Barton [9] for the acanthocephalan *Serrasentis sagittifer* (Linton, 1889) and the nematode *Digitiphilometroides marinus* (Moravec et de Buron, 2009), respectively, both parasitizing the marine fish (cobia) *Rachycentron canadum* (Linnaeus). Also the nematode *Piscicapillaria bursata* Moravec et Barton, 2019, a parasite of hammerhead sharks (*Sphyrna* spp.) described from off Australia [10] has recently been recorded from hammerheads off the Atlantic coast of South Carolina, USA [11].

### *Cucullanus* sp.

Host: Narrow-lined puffer *Arothron manilensis* (Marion de Procé) (Tetraodontidae, Tetraodontiformes).

Site of infection: Intestine.

Locality: Near Passe de Dumbéa, off Nouméa, New Caledonia (collected 22 November 2007).

Prevalence, intensity and details about fish: 1 fish infected/2 fish examined; 1 nematode; the infected fish (JNC2422) was 272 mm in fork length and 400 g in weight.

Deposition of voucher specimen: Muséum National d’Histoire Naturelle, Paris, France (MNHN JNC2422).

#### Remarks

Species identification was impossible, because only a single, not well-preserved female specimen was collected from this host. To date, only five nominal species of *Cucullanus* have been reported from tetraodontiform fishes, of which only *C*. *dodsworthi* Barreto, 1922 is known from hosts of the Tetraodontidae from Atlantic waters of Brazil, Mexico and Africa [6], whereas *C*. *bourdini*, *C*. *brevicaudatus* Pereira, Vieira et Luque, 2014, *C*. *hansoni* and *C*. *longipapillatus* Olsen, 1952 were reported from fishes of the Balistidae in French Polynesia, from off the Atlantic coast of Brazil, off Hawaii and New Caledonia, and off Hawaii, respectively [8, 16, 19, 21]. However, Moravec and Justine [16] considered the record of *C*. *bourdini*, a parasite of perciform fishes, from *Balistapus undulatus* (Park) (Balistidae) by Morand and Rigby [8] as probably based on misidentification. In view of a certain degree of host specificity of cucullanids, the present *Cucullanus* sp. from *A*. *manilensis* may belong to an undescribed new species.

### *Dichelyne* (*Cucullanellus*) *branchiostegi* (Yamaguti, 1941) Petter, 1974 Figure 16

Syn.: *Cucullanellus branchiostegi* Yamaguti, 1941.

**Figure 16:**
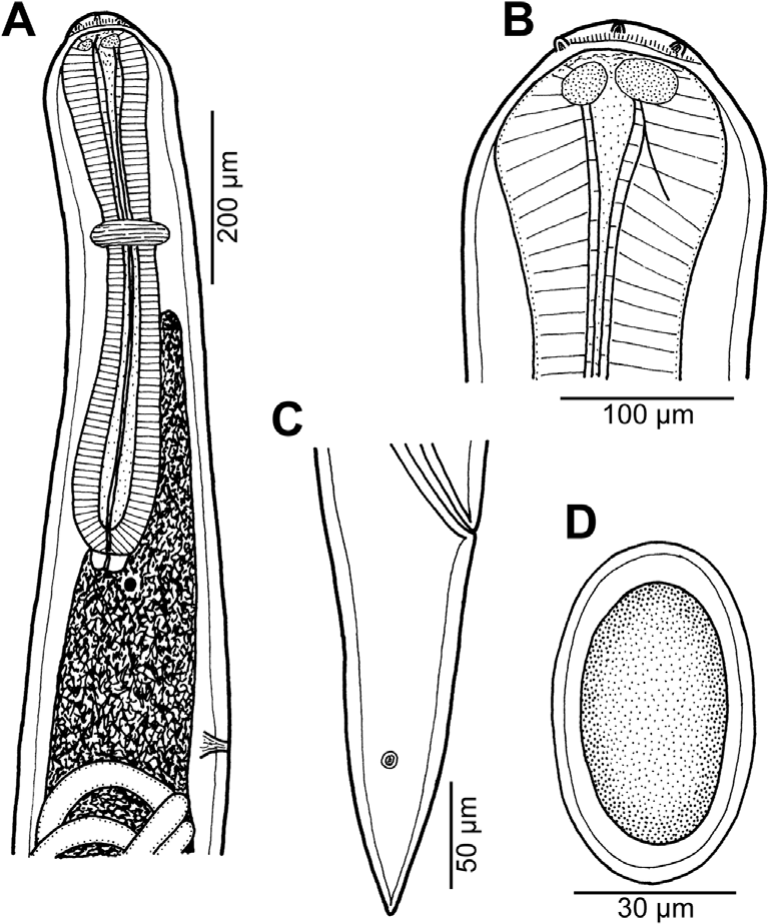
*Dichelyne* (*Cucullanellus*) *branchiostegi* (Yamaguti, 1941) from *Branchiostegus wardi*, gravid female. (A) Anterior end of body, lateral view; (B) cephalic end, lateral view; (C) tail, lateral view; (D) egg.

Host: Ward’s tilefish *Branchiostegus wardi* Whitney, 1932 (Malacanthidae, Perciformes).

Site of infection: Intestine.

Locality: External slope off Récif Kué, off Nouméa, New Caledonia, 22°35′707 S, 166°30′387 E (JNC2454).

Prevalence, intensity and details about fish: 1 fish infected/3 fish examined; 3 nematodes; the infected fish was 360 mm in fork length and 569 g in weight.

Deposition of voucher specimens: Muséum National d’Histoire Naturelle, Paris, France (MNHN JNC2454).

#### Description

*Female* (2 gravid specimens; measurements of 1 nongravid specimen in parentheses): Medium-sized nematodes with whitish, elongate body. Body length 6.87–7.05 (5.83) mm, maximum width at region of vulva 326–354 (258); width at level of oesophastome 150–163 (122), at mid-length of oesophagus 177–204 (163). Cuticle thick. Cephalic end dome-shaped in lateral view. Oral aperture surrounded by raised narrow membranous ala (collarette) supported by row of numerous minute basal teeth. Four submedian cephalic papillae and pair of lateral amphids present. Oesophagus muscular, 680–694 (625) long, representing 10 (11)% of body length, expanded at anterior end to form pseudobuccal capsule (oesophastome) 122–163 (136) long and 122–136 (108) wide; posterior part of oesophagus also expanded, 109–122 (95) wide; minimum width of oesophagus 54–68 (54) (Figs. 16A, 16B). Nerve ring encircles oesophagus at 41–42 (43)% of oesophagus length, at 286 (272) from anterior extremity. Ventral intestinal caecum 313 (82) long and 41–54 (41) wide, extending anteriorly to short distance posterior to level of nerve ring (Fig. 16A). Deirids situated near level of oesophago-intestinal junction, at 680–721 (734) from anterior end of body (Fig. 16A). Excretory pore situated some distance posterior to end of oesophagus, at 952–1,047 (966) from anterior extremity (Fig. 16A). Vulva postequatorial, 3.97–4.08 (3.26) mm from anterior extremity, at 56–59 (56)% of body length; vulval lips not elevated. Vagina directed anteriorly from vulva. Eggs oval, size 60–66 × 42–45 (Fig. 16D). Tail conical, pointed, 190 (177) long; lateral phasmids present, situated at posterior half of tail (Fig. 16C).

#### Remarks

Only female specimens of this nematode species were collected from *B*. *wardi*. Their general morphology, including the characteristic shapes of the cephalic end and oesophagus or the location of deirids, as well as all measurements are very close to those of females of *D*. (*C*.) *branchiostegi* [28]. Since the latter species was described from the congeneric fish host (*Branchiostegus japonicus* (Houttuyn)) in the Pacific region (Sea of Japan), the present nematodes from *B*. *wardi* are considered to belong to this species.

*Dichelyne* (*C*.) *branchiostegi* (reported as *Cucullanellus branchiostegi*) was originally described by Yamaguti [28] from *B*. *japonicus* from off Japan (Obama, Fukui Province) and has not been recorded since. Therefore, the present finding of *D*. (*C*.) *branchiostegi* in *B*. *wardi* off New Caledonia represents new host and geographical records.

### *Dichelyne* (*Cucullanellus*) *bodiani* Moravec et Justine, 2019

urn:lsid:zoobank.org:act:B0DCCA84-626C-4F25-BEDB-97CBBE48C51C

Hosts: Golden-spot hogfish *Bodianus perditio* (Quoy et Gaimard) and *Bodianus busellatus* Gomon (Labridae, Perciformes).

Site of infection: Intestine.

Localities: *B*. *perditio*: fish JNC2165, near Récif Toombo, off Nouméa, New Caledonia, 2 May 2007; fish JNC2167, same locality, 15 May 2007; fish JNC2939, same locality, 20 May 2009; *B*. *busellatus*, fish JNC3073, external reef near Passe de Dumbéa, off Nouméa, New Caledonia, collected 16 October 2009.

Prevalence, intensity and details about fish: *B*. *perditio*: 3 fish infected/50 fish examined; 1–15 nematodes; the infected fish were 346–500 mm in fork length and 654–2400 g in weight; a photograph of an infected fish has been deposited in Wikimedia as https://commons.wikimedia.org/wiki/File:Bodianus_perditio_JNC2165_with_colour_chart.JPG. *B*. *busellatus*: 1 fish infected/4 fish examined; 1 nematode; the infected fish was 236 mm in length and 249 g in weight; a photograph of the infected fish has been deposited in Wikimedia as https://commons.wikimedia.org/wiki/File:Bodianus_busellatus_JNC3073_-_with_colour_chart.JPG.

Deposition of voucher specimens: Muséum National d’Histoire Naturelle, Paris, France (MNHN JNC2165A, JNC2167C, JNC2939, JNC3073).

#### Remarks

This species has already been described by Moravec and Justine [16] based on specimens collected from *B*. *perditio* off New Caledonia. The present finding of *D*. (*C*.) *bodiani* in *B*. *busellatus* represents a new host record.

### *Dichelyne* (*Cucullanellus*) sp. Figure 17

Host: Golden-spot hogfish *Bodianus perditio* (Quoy et Gaimard) (Labridae, Perciformes).

**Figure 17:**
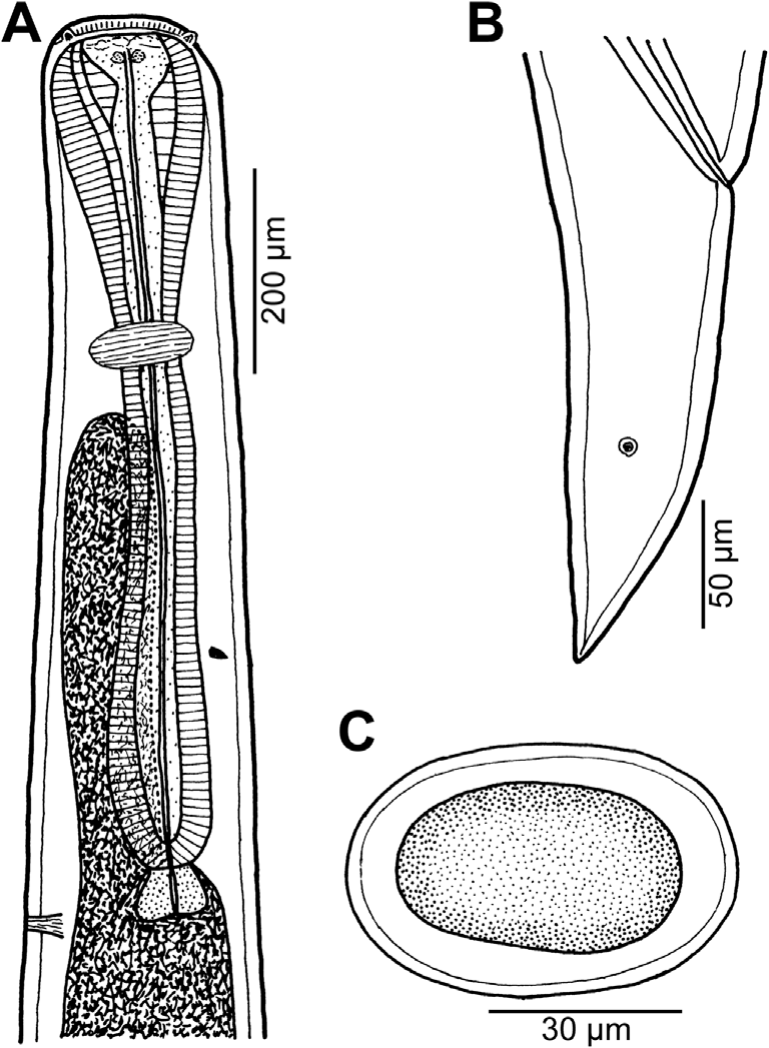
*Dichelyne* (*Cucullanellus*) sp. from *Bodianus perditio*, gravid female. (A) Anterior end of body, lateral view; (B) tail, lateral view; (C) egg.

Site of infection: Intestine.

Localities: Passe de Boulari (JNC1633C, collected 9 November 2005) and Récif Toombo (JNC2165B, collected 15 May 2007), New Caledonia.

Prevalence and intensity: 2 fish infected/50 fish examined; 1–2 nematodes.

Deposition of voucher specimens: Muséum National d’Histoire Naturelle, Paris, France (MNHN JNC1633C, JNC 2165B).

#### Description

*Female* (3 gravid specimens): Medium-sized nematodes with whitish, elongate body. Body length 8.20–11.19 mm, maximum width 272–354; width at level of oesophastome 177–204, at mid-length of oesophagus 218–231. Cuticle thick. Oral aperture surrounded by raised narrow membranous ala (collarette) supported by row of numerous minute basal teeth. Four submedian cephalic papillae and pair of lateral amphids present. Oesophagus muscular, 925–1,142 long, representing 10–11% of body length, expanded at anterior end to form pseudobuccal capsule (oesophastome) 245–326 long and 163–177 wide; posterior part of oesophagus also expanded, 109–136 wide; minimum width of oesophagus 68–82 (Fig. 17A). Nerve ring encircles oesophagus at 35–36% of oesophagus length, at 326–394 from anterior extremity. Ventral intestinal caecum 517–625 long and 68–136 wide, extending anteriorly nearly to level of nerve ring (Fig. 17A). Deirids situated approximately at level of mid-length of caecum, 694–884 from anterior end of body (Fig. 17A). Excretory pore situated short distance posterior to end of oesophagus, at 938–1200 from anterior extremity (Fig. 17A). Vulva postequatorial, 5.02–6.64 mm from anterior extremity, at 59–63% of body length; vulval lips not elevated. Vagina directed anteriorly from vulva. Eggs oval, size 60–69 × 36–48 (Fig. 17C). Tail conical, 190–218 long, with pointed tip; lateral phasmids present, situated at posterior half of tail (Fig. 17B).

#### Remarks

Altogether three gravid females of this form were recorded from two specimens of *B*. *perditio*, in one of them in a co-infection with *D*. (*C*.) *bodiani*. Their general morphology, such as the location of the excretory pore and deirids in relation to the oesophago-intestinal junction, the shape of the oesophagus or the anterior extent of the caecum, is similar to the latter species. However, they differ from gravid females of *D*. *bodiani* mainly in having a conspicuously larger body (body length 8.20–11.19 mm *vs.* 2.46–3.32 mm), a longer oesophagus (925–1142 μm *vs.* 639–707 μm) representing 10–11% (*vs.* 21–26%) of the entire body length and a longer tail (190–218 μm *vs.* 105–141 μm) with phasmids situated approximately in 2/3 (*vs.* approximately in the middle) of its length. On the other hand, the size of eggs is much the same in both these forms (60–69 × 36–48 μm and 60–72 × 42–48 μm).

Since no males of a comparable size to that of the present *Dichelyne* sp. females were found in *B*. *perditio* (the males of *D*. *bodiani* are only 2.26–3.13 mm long), it is impossible to decide on the basis of morphological features whether these nematodes (*Dichelyne* sp.) also belong to *D*. *bodiani* or they represent a different congeneric species. The use of molecular methods might prove to be helpful in solving this problem.
